# The Basicity Makes the Difference: Improved Canavanine-Derived Inhibitors of the Proprotein Convertase Furin

**DOI:** 10.1021/acsmedchemlett.0c00651

**Published:** 2021-02-09

**Authors:** Thuy Van Lam van, Miriam Ruth Heindl, Christine Schlutt, Eva Böttcher-Friebertshäuser, Ralf Bartenschlager, Gerhard Klebe, Hans Brandstetter, Sven O. Dahms, Torsten Steinmetzer

**Affiliations:** † Institute of Pharmaceutical Chemistry, Philipps University, Marbacher Weg 6, 35032 Marburg, Germany; ‡ Institute of Virology, Philipps University, Hans-Meerwein-Strasse 2, 35043 Marburg, Germany; § Department of Infectious Diseases, Molecular Virology, Heidelberg University and German Center for Infection Research, Heidelberg Partner Site, Im Neuenheimer Feld 344, 69120 Heidelberg, Germany; ∥ Department of Biosciences, University of Salzburg, Billrothstrasse 11, 5020 Salzburg, Austria

**Keywords:** furin inhibitors, proprotein convertases, canavanine, crystal structure analysis, proteolytic activation of viruses

## Abstract

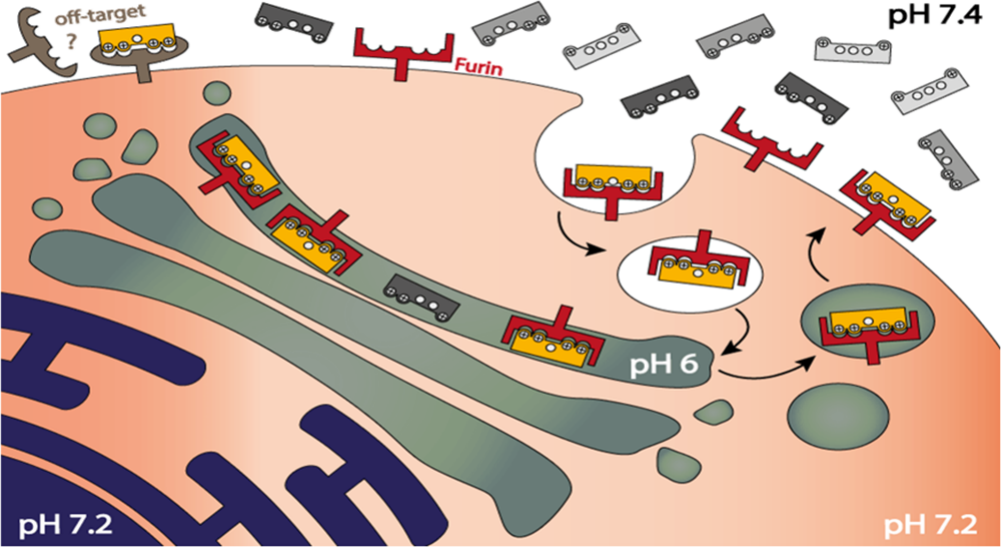

Furin activates numerous viral glycoproteins, and its inhibition prevents virus replication and spread. Through the replacement of arginine by the less basic canavanine, new inhibitors targeting furin in the trans-Golgi network were developed. These inhibitors exert potent antiviral activity in cell culture with much lower toxicity than arginine-derived analogues, most likely due to their reduced protonation in the blood circulation. Thus, despite its important physiological functions, furin might be a suitable antiviral drug target.

Furin belongs to the basic proprotein convertases (PCs), a family of seven Ca^2+^-dependent human subtilisin-like serine proteases. It activates a huge number of proproteins at characteristic multibasic recognition sequences, including proforms of hormones, enzymes, adhesion proteins, and various receptors.^1,2^ Given these important physiological functions, the whole body knockout of furin in mice is not tolerated and causes embryonic lethality 11 days after gestation. In contrast, an inducible liver-specific knockout in adult mice is well-tolerated, and no morphological abnormalities were found.^3^ As host protease, furin also activates various toxins of pathogenic bacteria as well as surface glycoproteins of numerous furin-dependent viruses. The cleavage of these viral glycoproteins is essential to ensure cell entry and fusion competence of virus particles, thus contributing to their pathogenicity.^4^ Therefore, furin emerged as a potential target for the development of broad-spectrum antivirals, at least for a short-term administration during acute infections. Based on sequence analysis^5^ and modification of the furin cleavage site,^6^ it was recently suggested that furin also contributes to the activation of the spike protein of the new SARS-coronavirus 2 (SARS-CoV-2) at the S1/S2 site, although a second cleavage by the trypsin-like serine protease TMPRSS2 at the S2′ site is required to unmask the fusion peptide.^7,8^

With our previously described 4-amidinobenzylamide (Amba) derived inhibitor **1**(9) and its analogues, we could inhibit the replication of numerous furin-dependent viruses in cell culture, like highly pathogenic bird flu strains H5N1 and H7N1,^10,11^ Chikungunya virus,^12^ West Nile and Dengue-2 virus,^13^ mumps virus,^14^ or respiratory syncytial virus (RSV).^15^ Although these inhibitors revealed only a negligible toxicity in all used cell cultures up to concentrations of 50 μM, they exhibited a significant toxicity in mice. While an intraperitoneal dose of 2.5 mg/kg of inhibitor **1** was accepted, all mice died within the first hour at the next higher dose of 5 mg/kg.^13^ A significant toxicity in mice for structurally related Amba-derived furin-like PC inhibitors was also reported by a different group.^16,17^ Because of previously or presently approved benzamidine derivatives like the prodrugs of melagatran and dabigatran or pentamidin, we assumed that the toxicity of compound **1** is not simply caused by the P1 Amba anchor, but also by the presence of the three additional strongly basic guanidine groups. This was confirmed by deletion or replacements of the individual guanidine and amidine moieties, which all provided less toxic analogues.^13^ However, most of these compounds exhibited a considerably weaker inhibitory potency against furin in enzyme kinetic measurements and strongly reduced or complete lack of any antiviral activity in infected cell cultures. Only the replacement of the P2 Arg in inhibitor **1** by Lys in case of compound **2** was tolerated and provided a slightly less toxic compound.^13^

This also raised the question of whether toxicity in mice is either generally caused by the inhibition of the physiological relevant furin itself, which activates most viral glycoproteins and physiological substrates in the trans-Golgi-network (TGN),^1,2^ or by addressing a different, so far unknown off-target in the blood circulation. For an antiviral effect through an effective inhibition of furin, the P2 and P4 side chains of substrate analogue inhibitors must be protonated in the TGN at a pH close to 6.0.^18,19^ Furthermore, we assumed that only the same completely protonated inhibitor species can address the hypothetical off-target in the circulation at a pH around 7.4. Therefore, we propose the hypothesis that this pH difference can be utilized to develop compartment-specific furin inhibitors by replacing the strongly basic P2 and P4 arginines with a residue that is almost completely protonated and positively charged under slightly acidic conditions in the TGN while only partially charged in the circulation and extracellular space. This strategy provided improved furin inhibitors with a stronger antiviral activity in cell culture and reduced toxicity in mice at the same time.

## Inhibitor Design

A drastic drop in the p*K*_a_ value of alkyl guanidines can be achieved by replacing the methylene group next to the guanidine with oxygen. Such oxyguanidine groups were incorporated in various thrombin inhibitors to improve their bioavailability.^20,21^ Based on this strategy, we prepared a new inhibitor series (Table 1) by replacing the P2 and/or P4 residues of inhibitors **1** and **2** by canavanine (Cav). Canavanine is an unusual amino acid containing a weakly basic oxyguanidine group with a p*K*_a_ value of 7.01.^22,23^ It should be largely protonated at pH 6.0 suitable for the inhibition of furin in the TGN, whereas it is only partially protonated at pH 7.4, thereby reducing the concentration of the completely protonated species addressing the unknown off-target in the circulation and improving the bioavailability. For instance, based on the Henderson–Hasselbalch equation, it can be roughly calculated for compound **8**, which contains two independently protonatable Cav residues, that at pH 6.0, approximately 80% of the inhibitor exists as the fully 4-fold protonated species, while at pH 7.4, it is only approximately 9%.

**Table 1:**
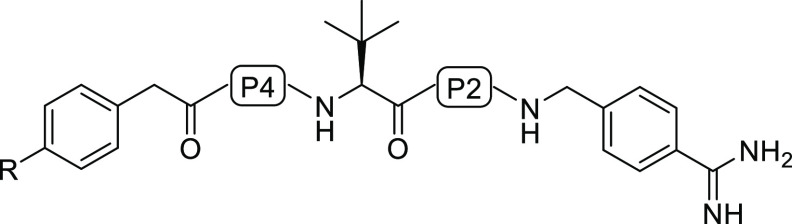

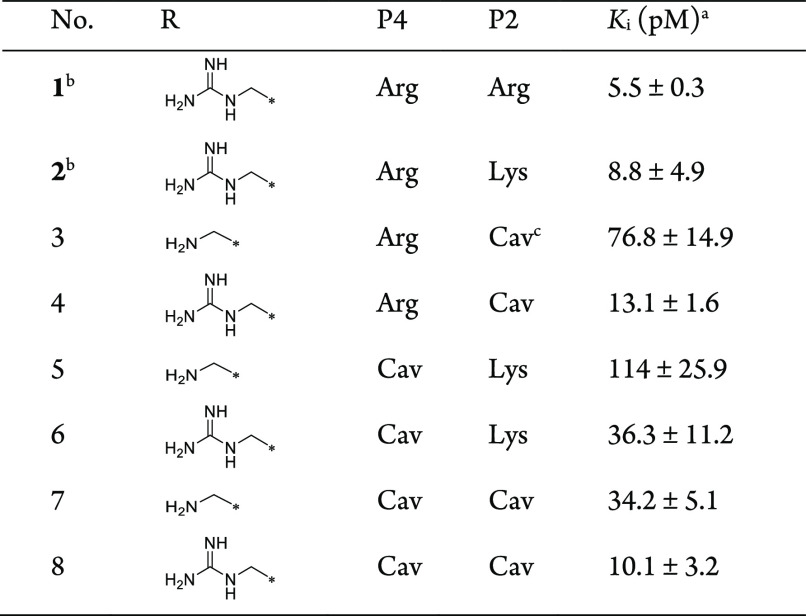
Structures and Potencies of the Synthesized Furin Inhibitors

a The assay was performed using recombinant soluble human furin and the fluorogenic substrate Phac-Arg-Val-Arg-Arg-AMC.^9^

b The reference inhibitors **1**(9) and **2**(13) were described previously.

c
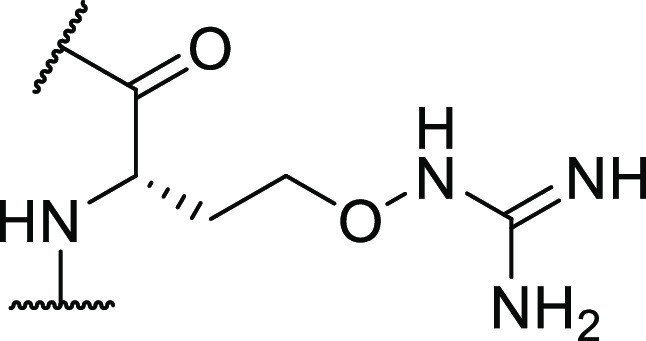

The replacement of the P2 Arg in inhibitor **1** provided the Cav derivative **4**, which possesses a 2.4-fold reduced potency in the enzyme kinetic assay, which was performed under our standard conditions at pH 7.0,^9^ where the Cav side chain should be incompletely protonated. The aminomethyl derivative **3** was prepared as an additional analogue lacking the guanidine substitution on the P5 phenylacetyl (Phac) group, although we knew from previous studies that this modification leads to a slightly reduced furin inhibition and weaker antiviral activity in cell culture.^9,10^ Compound **6** is the P4 Cav derivative of inhibitor **2**, whereas compounds **7** and **8** contain two Cav residues in the P2 and P4 position. Especially compounds **4** and **8** are still very potent furin inhibitors with *K*_i_ values <15 pM.

Notably, the replacement of the P2 and P4 Arg residues in inhibitor **1** with Cav further reduces the inhibitory potency of this compound type against trypsin. While compound **1** is still a relatively effective trypsin inhibitor possessing a *K*_i_ value of 52 nM,^24^ the affinity of inhibitor **8** is 10-fold reduced (*K*_i_ for trypsin 690 nM). Furthermore, a negligible potency (*K*_i_ > 3 μM) was found for the other tested trypsin-like serine proteases thrombin, factor Xa, and plasmin (Table S3).

## Crystal Structure Determination

**Figure 1:**
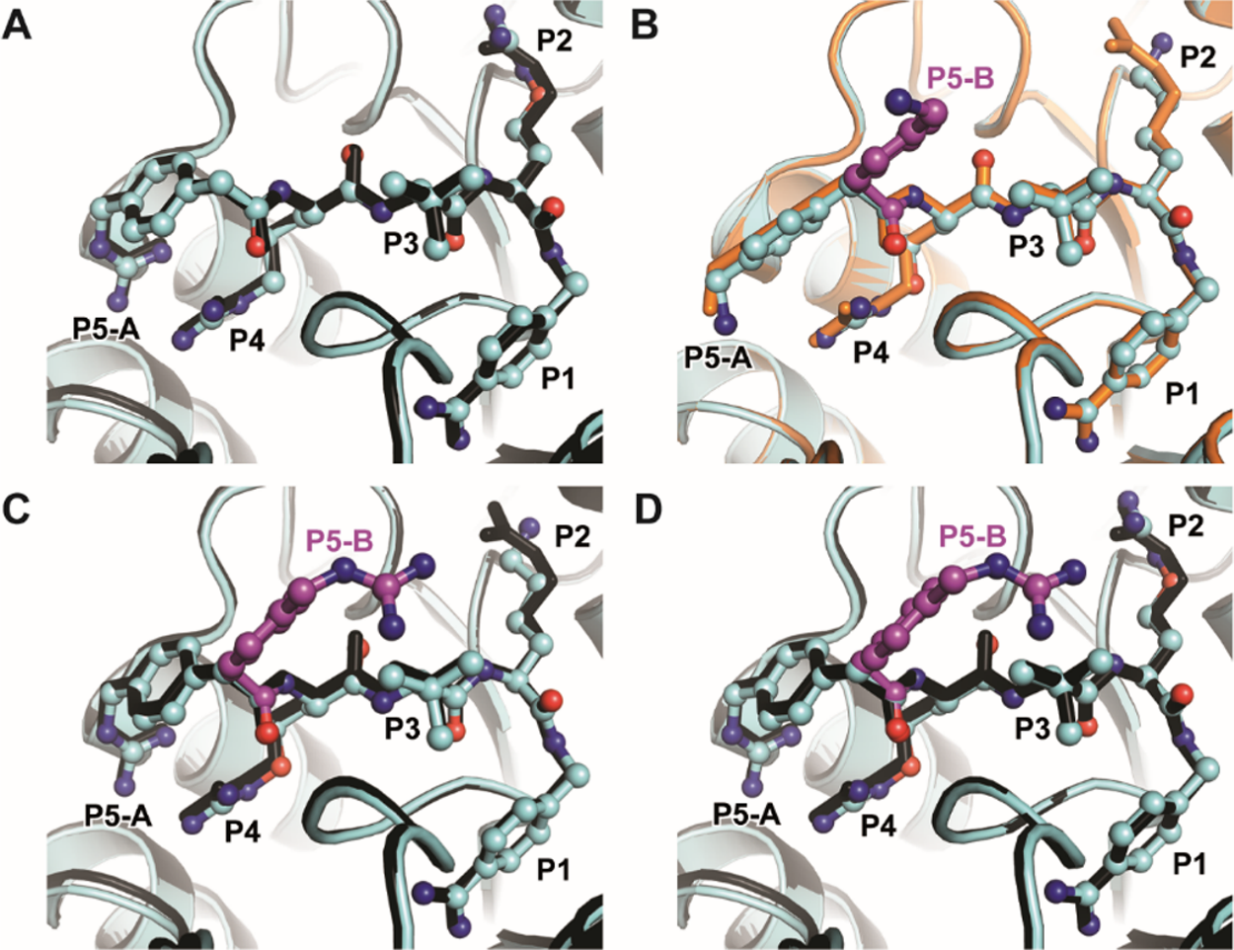
Crystal structures of furin in complex with Cav-based inhibitors. Furin and the inhibitors are shown as cartoon and ball-and-stick models, respectively (cyan). (A, C, D) Furin in complex with inhibitors **4** (A), **6** (C), and **8** (D) all superimposed to the structure of furin (cartoon, black) in complex with analogue **1**(9) (stick model, black). (B) Furin in complex with inhibitor **5** superimposed to the structure of furin (cartoon, black) in complex with 4-aminomethyl-phenylacetyl-Arg-Tle-Arg-Amba^26^ (stick model, orange). (A–D) The major conformation of the P5 residue is labeled as P5-A, alternative conformations are shown in magenta (P5-B).

Due to the sp^3^ hybridization of the oxygen, we expected that the side chain of Cav can adopt an identical geometry like arginine in the P4 position, which is a prerequisite for an efficient furin inhibition by substrate analogue structures. This assumption could be proven by crystal structures of inhibitors **4**–**6** and **8**, which were determined after soaking the compounds into crystals of ligand-free furin.^25^ Resolutions between 1.7–2.0 Å have been observed for all complexes, as well as good stereochemistry and R-factors (Table S2). In all crystal structures, furin was found to adopt the “ON”-state enabling a canonical protease-ligand interaction pattern (Figure 1A–D). This includes a specific change of the conformation of the alignment template and of the catalytic triad triggered by ligand binding.^25^ The observed Cα-RMSD values between the structures are lower than 0.1 Å, indicating high overall similarity.

An identical side chain conformation was observed for the P2 Cav-derived inhibitors **4** and **8** (Figure 1A, D) when compared with the previously determined crystal structure of the P2 arginine analogue **1** in furin.^9^ The same applies for the incorporation of Cav in the P4 position as found in the complexes with inhibitors **5**, **6**, and **8** (Figure 1B, C).

**Figure 2:**
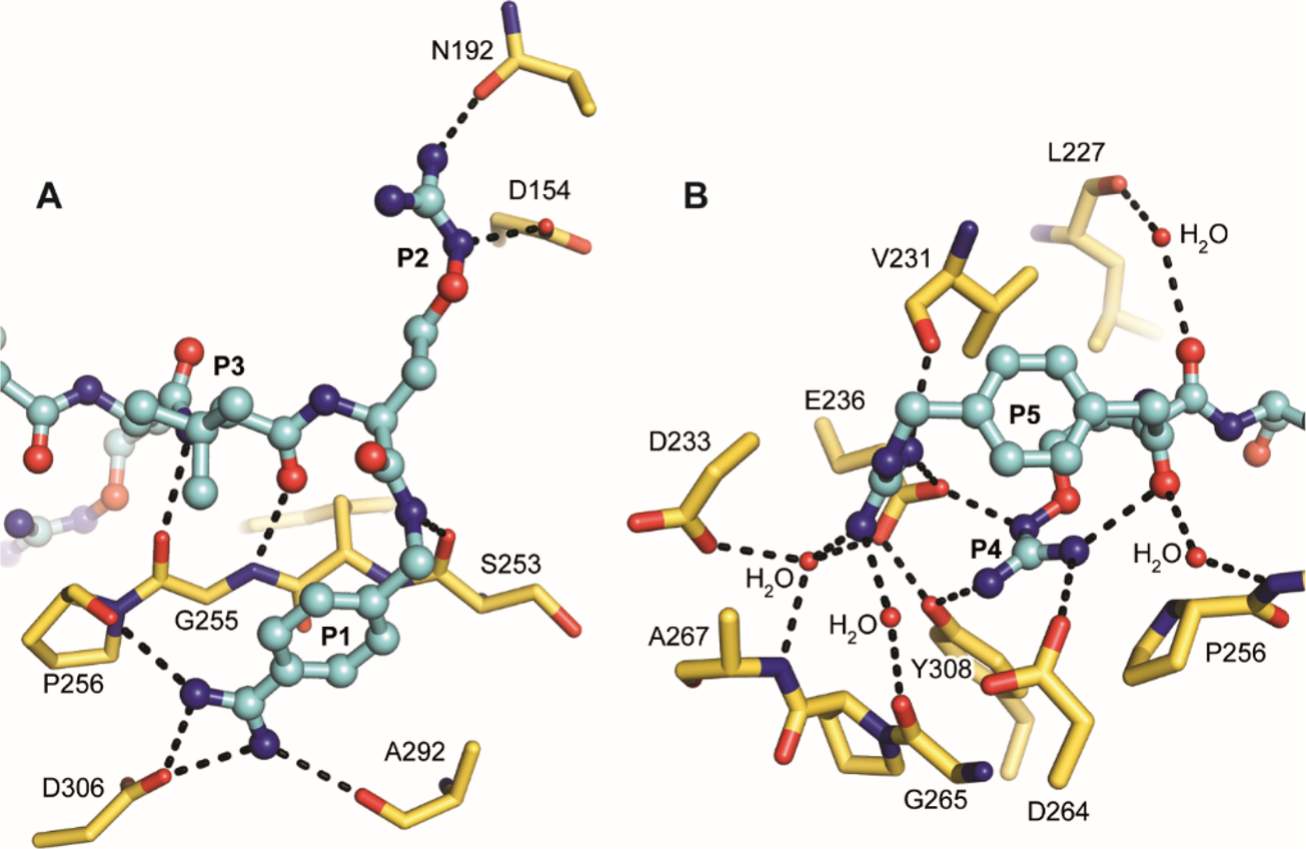
Polar interactions of inhibitor **8** (ball-and-stick model with carbons in cyan) with furin shown as a stick model with yellow carbons, hydrogen bonds are shown as black dashed lines. (A) Polar interactions of the inhibitor’s P3–P1 segment. The P1 amidine forms a salt bridge to Asp306 and two additional hydrogen bonds with the carbonyl oxygens of Pro256 and Ala292. The P1 backbone NH binds to the carbonyl oxygen of Ser253. The oxyguanidino moiety of the P2 residue interacts with the carboxyl of Asp154 and the side chain of Asn192, respectively. The P3 backbone makes antiparallel beta-sheet-like hydrogen bonds with Gly255. (B) Polar interactions of the inhibitor’s P5–P4 segment. The P4 Cav side chain contacts Asp264, Glu236, and Tyr308, respectively, whereas the P4 carbonyl binds to Leu227 via a water molecule. The P5 guanidino group makes electrostatic interactions with the carboxyl of Glu236 and a hydrogen bond to the carbonyl of Val231. An intramolecular hydrogen bond is formed between the P4 side chain and P5 carbonyl oxygen. Water bridges interactions are found between the P5 carbonyl oxygen and Glu257 NH and from the P5 guanidino group to Gly265, Asp233, and Ala267. It is noteworthy that these interactions of the P5 guanidino group are only observed for conformation A, whereas conformation B is not involved in polar contacts.

Interestingly, the P4-Cav was found to affect the binding of the N-terminal P5-group in the case of inhibitors **5**, **6**, and **8**, which was found to adopt two prevailing conformations with similar occupancies (Figure 1B–D). In comparison to their P1–P4 segments, we observed a less well-defined electron density map for the conformation P5-A of these inhibitors with occupancies of 0.5, 0.5, and 0.57, respectively (Figure S1B–D). It is noteworthy that the minor conformation P5-B does not mediate specific attractive interactions with furin. This effect was observed for both the aminomethyl-Phac (inhibitor **5**, Figure 1B) and the guanidinomethyl-Phac group (inhibitors **6** and **8**, Figure 1C, D) at the P5 position. In contrast, if arginine was present at P4 (inhibitor **4**, Figure 1A), P5 was found in its typical conformation known from inhibitor **1**(9) as indicated by the well-defined electron density map (Figure S1A). Interestingly, we observed either no deteriorating effect (inhibitor **4** vs **8**) on the *K*_i_ value or only a moderate drop in inhibitory potency (inhibitor **2** vs **6**) due to these alternative P5 conformations caused by the exchange of Arg by Cav at P4. This observation was unexpected because in previous studies we found a strong contribution of the guanidinomethyl-substituted P5 group of up to two orders of magnitude on the *K*_i_ value compared with the unsubstituted Phac derivative.^10^ A potential loss of binding affinity at P5, as suggested by the distorted P5 residue in the structures with inhibitors **5**, **6**, and **8**, might be compensated by a stronger contribution of P4-Cav, consistent with an increased binding affinity of compound **7** compared with that of inhibitor **3**. The Cav side chain shows a different charge distribution compared to arginine because of the electronegative oxygen atom of its oxyguanidino moiety, which might fit better to the S4 pocket of furin. Another explanation might be an impact of P4 Cav to the binding thermodynamics of P5. The alternative occupation of the P5 residue indicates a reduced contribution of hydrogen bonds and electrostatic interactions and thus a reduction of the binding enthalpy. However, the flexibility of the P5 residue is increased in this setting and might compensate the loss of enthalpy by a gain in entropy while keeping the Gibbs free energy virtually unchanged. Figure 2 shows the polar contacts of inhibitor **8** in complex with furin.

## Antiviral Activity

**Figure 3:**
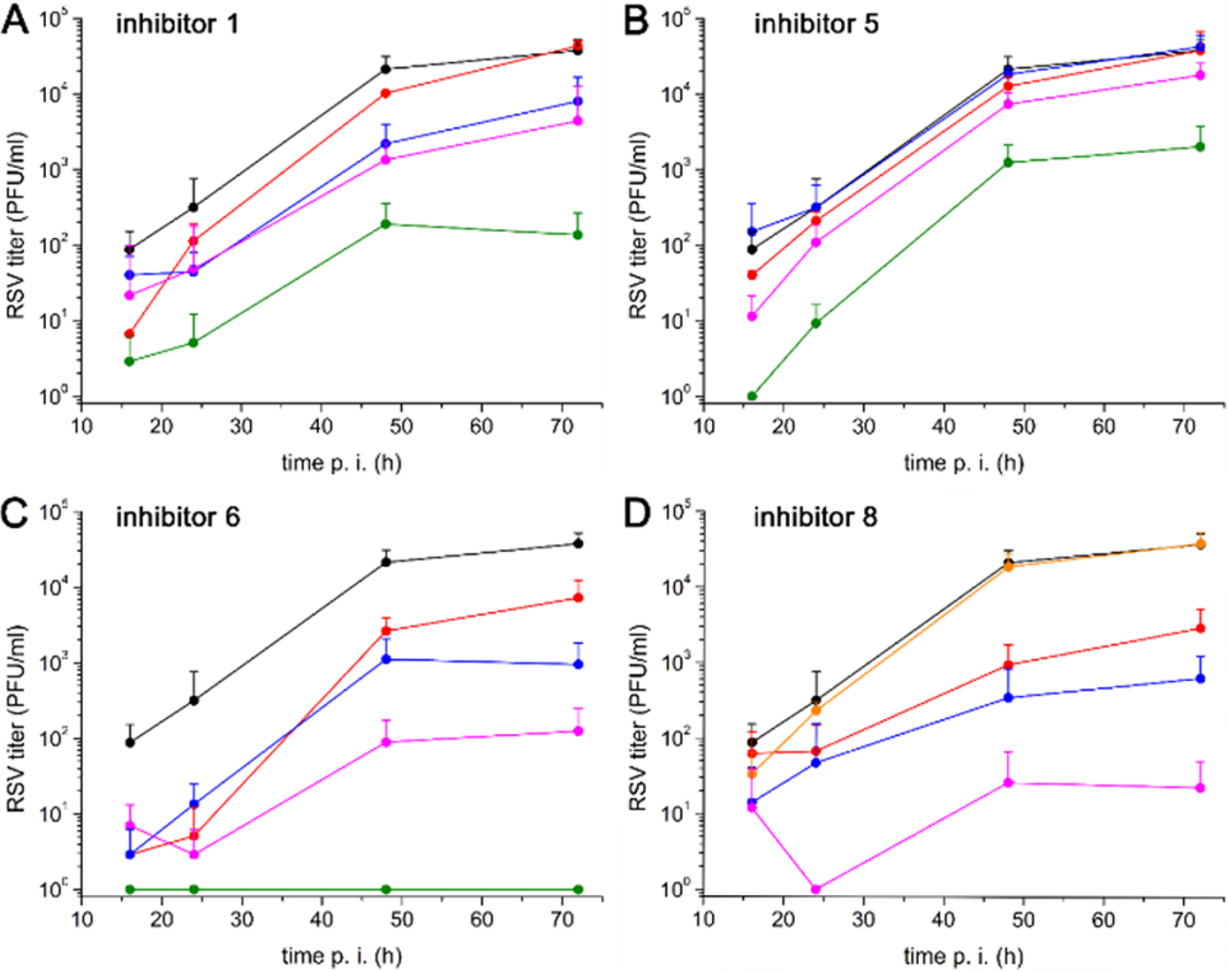
Multicycle replication of RSV in A549 cells. Cells were inoculated with an MOI of 1 for 1 h. After washing, the cells were incubated with various concentrations (control without inhibitor (black), 10 μM (green), 2.5 μM (pink), 1.0 μM (blue), and 0.5 μM (red) of the reference inhibitor **1** (A) and the canavanine-containing analogues **5** (B) and **6** (C)). In case of inhibitor **8** (D), the 10 μM concentration was replaced with a 0.1 μM (orange) concentration. At 16, 24, 48, and 72 h post infection, cell supernatants were collected, and viral titers ± standard deviation (*n* = 3) were determined by plaque assay (plaque-forming units (PFU) per mL).

Among many viral glycoproteins,^2,27^ furin activates the precursor of the fusion protein F of RSV at two multibasic sequences. One of them is directly located at the N-terminal side of the fusion peptide (Lys131-Lys-Arg-Lys-Arg-Arg136↓) and the second 27 residues upstream at the Arg106-Ala-Arg-Arg109↓ segment.^28,29^ Both cleavages and the concomitant release of a 27-mer peptide are essential for the fusion capacity of F and productive infection of target cells. RSV infects nearly all children by two years of age and in some cases, especially in very young children, this can lead to airway inflammation, bronchiolitis, and pneumonia. Worldwide, approximately 3.2 million hospitalizations leading to around 66 000 in-hospital deaths annually in infants younger than 5 years have been reported.^30,31^ The replication mechanism of RSV suggests a furin inhibition as potential antiviral therapy. Therefore, we investigated selected inhibitors in a multicycle replication assay in A549 human lung cancer cells infected with the RSV A2 strain, as reported previously for inhibitor **1**,^15^ which was used as a reference compound. Compared with inhibitor **1**, a reduced RSV inhibition was observed with compound **5**, possessing a 4-aminomethyl substitution on the P5 Phac group, while the 4-guanidinomethyl analogue **6** was slightly more potent than the reference inhibitor (Figure 3A–C). The strongest antiviral efficacy was found for inhibitor **8**. Based on initial tests, in which a complete inhibition of virus replication was found at 10 μM of inhibitor **8**, 0.1 μM was used as lowest concentration for this compound (Figure 3D).

**Figure 4:**
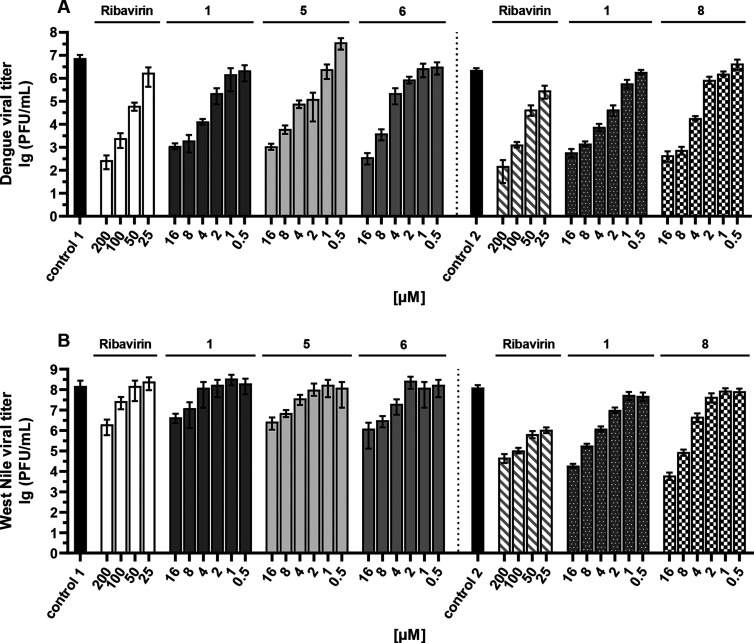
Infection of Huh-7 cells with DENV-2 (A) and WNV (B) in the presence of selected inhibitors. Cell culture supernatants were harvested 48 h postinfection, and virus titers ± standard deviation (*n* = 2) were determined by plaque assay in VeroE6 cells. The effects of the reference compounds **1** and ribavirin as well as details of the assay methods used have been described recently.^13,32^

Furthermore, the host protease furin activates the prM precursor of the membrane protein M of numerous pathogenic flaviviruses like Dengue, West Nile, Zika, Yellow fever, Japanese encephalitis, St. Louis encephalitis virus, and others. Furin cleavage of prM is essential to render virus particles infectious. Therefore, the antiviral activity of compounds **5** and **6** was tested in Huh-7 cells infected with dengue virus (strain 16681) or with West-Nile virus, as described previously.^13,32^ In a second experiment, inhibitor **8** was also included (Figure 4). Inhibitor **1** and the nucleoside analogue ribavirin were used as reference compounds. Also with these viruses, a significant antiviral potency was observed for inhibitors **5**, **6**, and **8** (Figure 4). For compound **1**, EC_50_ values of 0.76 and 0.91 μM were determined for the inhibition of DENV-2 and WNV replication, respectively. Slightly higher EC_50_ values of 1.50 μM (DENV-2) and 1.46 μM (WNV) were calculated for inhibitor **8**. For both compounds, the cytotoxicity in Huh-7 cells (CC_50_) is >50 μM (highest tested concentration). This provided a selectivity index (SI = CC_50_/EC_50_) >33 for compound **8** against both flaviviruses, the SI values for the reference inhibitor **1** are >65 (DENV-2) and >55 (WNV).

## Toxicity Studies

Three Cav-derived inhibitors, converted into their physiologically more acceptable hydrochlorides as described previously,^13^ were tested for toxicity after intraperitoneal (*ip*) treatment in mice. On the first day, the mice were treated with 2.5 mg/kg inhibitor and only, if all four mice survived, with the next higher dose always 24 h later (5, 10, or 15 mg/kg). In contrast to the reference inhibitors **1** and **2**, a reduced toxicity was found for all three Cav derivatives (Table 2). Together with the results from antiviral testing, where compounds **6** and **8** were nearly equipotent to inhibitor **1**, it can be concluded that the toxicity of this compound type does not correlate with the inhibition of intracellular furin, leading to an antiviral activity. So far, we cannot explain the exact mechanism leading to the reduced toxicity of the less basic Cav-containing inhibitor **8** compared to its isostructural Arg analogue **1**. Notably, similar plasma levels were obtained after intravenous treatment of rats with 1 mg/kg of both compounds. However, two of three rats died within 90 min after *iv* treatment with inhibitor **1** at this concentration (data not shown), whereas compound **8** was well-accepted in all three rats without any signs of side effects, which is a significant advantage compared to our previous inhibitors. Hence, we suspect that a different, so far unknown off-target must be responsible for the toxicity of these benzamidine derivatives, which confirms our previous results.^13^

For further characterization, the best compound **8** was investigated for its pharmacokinetic (PK) properties in rats. The inhibitor was given intravenously (1 mg/kg) and displayed a half-life of 0.9 h and a total body clearance of 442 mL/(h·kg). After intraperitoneal treatment (2.5 mg/kg), a half-life of 1.6 h, a total body clearance of 273 mL/(h·kg), and a maximal concentration (*C*_max_) of 3156 ng/mL after 0.5 h were determined. This provided plasma levels >1000 ng/mL (∼1.3 μM) over a period of approximately 3 h (Figure S6). Such a concentration is close to the range of the EC_50_ values determined for the inhibition with DENV-2 and WNV replication in cell culture studies.

**Table 2:** Toxicity Study in Mice

no.	tolerated dose (mg/kg)	number of deaths at next higher dose^a^
**1**^b^	2.5	4 of 4 at 5 mg/kg
**2**^b^	5	4 of 4 at 10 mg/kg
**5**	15	no higher dose tested
**6**	10	1 of 4 at 15 mg/kg
**8**	15	no higher dose tested

a In each group, four mice (two female and two male) were intraperitoneally treated.

b Published previously^13^

The replacement of arginine by canavanine provided a new series of highly potent furin inhibitors which exhibit a binding mode similar to that found previously in crystal structures of furin in complex with their arginine analogues. The tested compounds possess a significant antiviral activity against furin-dependent viruses, like RSV, WNV, and Dengue-2 virus. The strongest efficacy against RSV was found for inhibitor **8**. This compound also exhibits an efficient inhibition of WNV and Dengue-2 virus replication and possesses a considerably reduced toxicity in mice and rats compared to compound **1**.

Our results prove that the toxicity of these substrate–analogue furin inhibitors does not correlate with the strength of the intracellular furin inhibition. Despite its manifold physiological functions, this makes furin a suitable therapeutic target, especially for the short-term treatment of certain acute infectious diseases. As described recently, treatment with the furin inhibitor **8** also provided a significant reduction of the SARS-CoV-2 replication in infected Calu-3 cells due to the inhibition of the S1/S2 cleavage in its spike surface protein.^8^ Moreover, in previous studies using cell cultures infected with highly pathogenic avian influenza strains,^11^ we demonstrated a beneficial antiviral effect when the inhibitors of the host protease furin were used in combination with additional drugs addressing viral proteins. This strategy allowed a further reduction of the individual inhibitor concentrations while improving the antiviral efficacy. We are confident that the new and less toxic canavanine-derived furin inhibitors, like compound **8**, are suited for similar combination therapies, which should further reduce drug-related side effects *in vivo* and the rapid development of resistant virus strains.

## Supporting Information Available

Synthesis^13^ and analytical characterization of the inhibitors; protein crystallography with furin including the soaking of the inhibitors into ligand free crystals of furin;^25^ diffraction data collection, data processing, model building and refinement;^26^ conditions of the enzyme kinetic measurements; determination of the antiviral activity against RSV,^15^ Dengue-2 virus,^32^ and West Nile virus^32^ in infected cell cultures; toxicity studies in mice;^13^ and details of the PK study with inhibitor **8** in rats (PDF).

## Accession Codes

PDB IDs: 6YD7 (**4**), 6YD2 (**5**), 6YD3 (**6**), and 6YD4 (**8**).

## Abbreviations

Amba: 4-amidinobenzylamide
Cav: canavanine
DENV-2: Dengue virus serotype 2
*i**p*: intraperitoneal
*iv*: intravenous
MOI: multiplicity of infection
PC: proprotein convertase
Phac: phenylacetyl
RMSD: root mean square deviation
RSV: respiratory syncytial virus
TGN: trans-Golgi network
Tle: *tert*-leucine
WNV: West-Nile virus

## References

[ref1] Thomas G. Furin at the cutting edge: from protein traffic to embryogenesis and disease. Nat. Rev. Mol. Cell Biol. 2002, 3 (10), 753–66. 10.1038/nrm934. 12360192 PMC1964754

[ref2] Seidah N. G.; Prat A. The biology and therapeutic targeting of the proprotein convertases. Nat. Rev. Drug Discovery 2012, 11 (5), 367–83. 10.1038/nrd3699. 22679642

[ref3] Roebroek A. J.; Taylor N. A.; Louagie E.; Pauli I.; Smeijers L.; Snellinx A.; Lauwers A.; Van de Ven W. J.; Hartmann D.; Creemers J. W. Limited redundancy of the proprotein convertase furin in mouse liver. J. Biol. Chem. 2004, 279 (51), 53442–50. 10.1074/jbc.M407152200. 15471862

[ref4] Klenk H. D.; Garten W. Host cell proteases controlling virus pathogenicity. Trends Microbiol. 1994, 2 (2), 39–43. 10.1016/0966-842X(94)90123-6. 8162439

[ref5] Coutard B.; Valle C.; de Lamballerie X.; Canard B.; Seidah N. G.; Decroly E. The spike glycoprotein of the new coronavirus 2019-nCoV contains a furin-like cleavage site absent in CoV of the same clade. Antiviral Res. 2020, 176, 104742 10.1016/j.antiviral.2020.104742. 32057769 PMC7114094

[ref6] Walls A. C.; Park Y. J.; Tortorici M. A.; Wall A.; McGuire A. T.; Veesler D. Structure, Function, and Antigenicity of the SARS-CoV-2 Spike Glycoprotein. Cell 2020, 183, 1735 10.1016/j.cell.2020.11.032. 33306958 PMC7833104

[ref7] Hoffmann M.; Kleine-Weber H.; Schroeder S.; Krüger N.; Herrler T.; Erichsen S.; Schiergens T. S.; Herrler G.; Wu N. H.; Nitsche A.; Müller M. A.; Drosten C.; Pöhlmann S. SARS-CoV-2 Cell Entry Depends on ACE2 and TMPRSS2 and Is Blocked by a Clinically Proven Protease Inhibitor. Cell 2020, 181, 271 10.1016/j.cell.2020.02.052. 32142651 PMC7102627

[ref8] Bestle D.; Heindl M. R.; Limburg H.; Van Lam van T.; Pilgram O.; Moulton H.; Stein D. A.; Hardes K.; Eickmann M.; Dolnik O.; Rohde C.; Klenk H. D.; Garten W.; Steinmetzer T.; Böttcher-Friebertshäuser E. TMPRSS2 and furin are both essential for proteolytic activation of SARS-CoV-2 in human airway cells. Life science alliance 2020, 3 (9), e202000786 10.26508/lsa.202000786. 32703818 PMC7383062

[ref9] Hardes K.; Becker G. L.; Lu Y.; Dahms S. O.; Köhler S.; Beyer W.; Sandvig K.; Yamamoto H.; Lindberg I.; Walz L.; von Messling V.; Than M. E.; Garten W.; Steinmetzer T. Novel furin inhibitors with potent anti-infectious activity. ChemMedChem 2015, 10 (7), 1218–31. 10.1002/cmdc.201500103. 25974265

[ref10] Becker G. L.; Lu Y.; Hardes K.; Strehlow B.; Levesque C.; Lindberg I.; Sandvig K.; Bakowsky U.; Day R.; Garten W.; Steinmetzer T. Highly potent inhibitors of proprotein convertase furin as potential drugs for treatment of infectious diseases. J. Biol. Chem. 2012, 287 (26), 21992–22003. 10.1074/jbc.M111.332643. 22539349 PMC3381159

[ref11] Lu Y.; Hardes K.; Dahms S. O.; Böttcher-Friebertshäuser E.; Steinmetzer T.; Than M. E.; Klenk H. D.; Garten W. Peptidomimetic furin inhibitor MI-701 in combination with oseltamivir and ribavirin efficiently blocks propagation of highly pathogenic avian influenza viruses and delays high level oseltamivir resistance in MDCK cells. Antiviral Res. 2015, 120, 89–100. 10.1016/j.antiviral.2015.05.006. 26022200

[ref12] Hardes K.; Ivanova T.; Thaa B.; McInerney G. M.; Klokk T. I.; Sandvig K.; Künzel S.; Lindberg I.; Steinmetzer T. Elongated and Shortened Peptidomimetic Inhibitors of the Proprotein Convertase Furin. ChemMedChem 2017, 12 (8), 613–620. 10.1002/cmdc.201700108. 28334511 PMC5572662

[ref13] Ivanova T.; Hardes K.; Kallis S.; Dahms S. O.; Than M. E.; Künzel S.; Böttcher-Friebertshäuser E.; Lindberg I.; Jiao G. S.; Bartenschlager R.; Steinmetzer T. Optimization of Substrate-Analogue Furin Inhibitors. ChemMedChem 2017, 12 (23), 1953–1968. 10.1002/cmdc.201700596. 29059503

[ref14] Krüger N.; Sauder C.; Huttl S.; Papies J.; Voigt K.; Herrler G.; Hardes K.; Steinmetzer T.; Orvell C.; Drexler J. F.; Drosten C.; Rubin S.; Müller M. A.; Hoffmann M. Entry, Replication, Immune Evasion, and Neurotoxicity of Synthetically Engineered Bat-Borne Mumps Virus. Cell Rep. 2018, 25 (2), 312–320. 10.1016/j.celrep.2018.09.018. 30304672

[ref15] Van Lam van T.; Ivanova T.; Hardes K.; Heindl M. R.; Morty R. E.; Böttcher-Friebertshäuser E.; Lindberg I.; Than M. E.; Dahms S. O.; Steinmetzer T. Design, Synthesis, and Characterization of Macrocyclic Inhibitors of the Proprotein Convertase Furin. ChemMedChem 2019, 14 (6), 673–685. 10.1002/cmdc.201800807. 30680958

[ref16] Gagnon H.; Beauchemin S.; Kwiatkowska A.; Couture F.; D’Anjou F.; Levesque C.; Dufour F.; Desbiens A. R.; Vaillancourt R.; Bernard S.; Desjardins R.; Malouin F.; Dory Y. L.; Day R. Optimization of furin inhibitors to protect against the activation of influenza hemagglutinin H5 and Shiga toxin. J. Med. Chem. 2014, 57 (1), 29–41. 10.1021/jm400633d. 24359257

[ref17] Kwiatkowska A.; Couture F.; Levesque C.; Ly K.; Beauchemin S.; Desjardins R.; Neugebauer W.; Dory Y. L.; Day R. Novel Insights into Structure-Activity Relationships of N-Terminally Modified PACE4 Inhibitors. ChemMedChem 2016, 11 (3), 289–301. 10.1002/cmdc.201500532. 26751825

[ref18] Paroutis P.; Touret N.; Grinstein S. The pH of the secretory pathway: measurement, determinants, and regulation. Physiology 2004, 19, 207–15. 10.1152/physiol.00005.2004. 15304635

[ref19] Casey J. R.; Grinstein S.; Orlowski J. Sensors and regulators of intracellular pH. Nat. Rev. Mol. Cell Biol. 2010, 11 (1), 50–61. 10.1038/nrm2820. 19997129

[ref20] Kim K. S.; Moquin R. V.; Qian L.; Morrison R. A.; Seiler S. M.; Roberts D. G. M.; Ogletree M. L.; Youssef S.; Chong S. Preparation of Argatroban analog thrombin inhibitors with reduced basic guanidine moiety. Med. Chem. Res. 1996, 6 (6), 377–383.

[ref21] Tomczuk B.; Lu T.; Soll R. M.; Fedde C.; Wang A.; Murphy L.; Crysler C.; Dasgupta M.; Eisennagel S.; Spurlino J.; Bone R. Oxyguanidines: application to non-peptidic phenyl-based thrombin inhibitors. Bioorg. Med. Chem. Lett. 2003, 13 (8), 1495–8. 10.1016/S0960-894X(03)00125-2. 12668020

[ref22] Boyar A.; Marsh R. E. l-Canavanine, a paradigm for the structures of substituted guanidines. J. Am. Chem. Soc. 1982, 104 (7), 1995–1998. 10.1021/ja00371a033.

[ref23] Pajpanova T.; Stoev S.; Golovinsky E.; Krauss H. J.; Miersch J. Canavanine derivatives useful in peptide synthesis. Amino Acids 1997, 12 (2), 191–204. 10.1007/BF01386482. 10794131

[ref24] Löw K.; Hardes K.; Fedeli C.; Seidah N. G.; Constam D. B.; Pasquato A.; Steinmetzer T.; Roulin A.; Kunz S. A novel cell-based sensor detecting the activity of individual basic proprotein convertases. FEBS J. 2019, 286 (22), 4597–4620. 10.1111/febs.14979. 31276291

[ref25] Dahms S. O.; Arciniega M.; Steinmetzer T.; Huber R.; Than M. E. Structure of the unliganded form of the proprotein convertase furin suggests activation by a substrate-induced mechanism. Proc. Natl. Acad. Sci. U. S. A. 2016, 113 (40), 11196–11201. 10.1073/pnas.1613630113. 27647913 PMC5056075

[ref26] Dahms S. O.; Hardes K.; Steinmetzer T.; Than M. E. X-ray Structures of the Proprotein Convertase Furin Bound with Substrate Analogue Inhibitors Reveal Substrate Specificity Determinants beyond the S4 Pocket. Biochemistry 2018, 57 (6), 925–934. 10.1021/acs.biochem.7b01124. 29314830

[ref27] Klenk H. D.; Garten W. Activation cleavage of viral spike proteins. In Cellular receptors for animal viruses. Monograph 28, Wimmer E., Ed.; Gold Spring Harbor Laboratory Press: 1994; pp 241–280.

[ref28] Gonzalez-Reyes L.; Ruiz-Arguello M. B.; Garcia-Barreno B.; Calder L.; Lopez J. A.; Albar J. P.; Skehel J. J.; Wiley D. C.; Melero J. A. Cleavage of the human respiratory syncytial virus fusion protein at two distinct sites is required for activation of membrane fusion. Proc. Natl. Acad. Sci. U. S. A. 2001, 98 (17), 9859–64. 10.1073/pnas.151098198. 11493675 PMC55543

[ref29] Zimmer G.; Budz L.; Herrler G. Proteolytic activation of respiratory syncytial virus fusion protein. Cleavage at two furin consensus sequences. J. Biol. Chem. 2001, 276 (34), 31642–50. 10.1074/jbc.M102633200. 11418598

[ref30] Battles M. B.; McLellan J. S. Respiratory syncytial virus entry and how to block it. Nat. Rev. Microbiol. 2019, 17 (4), 233–245. 10.1038/s41579-019-0149-x. 30723301 PMC7096974

[ref31] Cockerill G. S.; Good J. A. D.; Mathews N. State of the Art in Respiratory Syncytial Virus Drug Discovery and Development. J. Med. Chem. 2019, 62 (7), 3206–3227. 10.1021/acs.jmedchem.8b01361. 30411898

[ref32] Kouretova J.; Hammamy M. Z.; Epp A.; Hardes K.; Kallis S.; Zhang L.; Hilgenfeld R.; Bartenschlager R.; Steinmetzer T. Effects of NS2B-NS3 protease and furin inhibition on West Nile and Dengue virus replication. J. Enzyme Inhib. Med. Chem. 2017, 32 (1), 712–721. 10.1080/14756366.2017.1306521. 28385094 PMC6445162

