# Supplementary material for: The Basicity Makes the Difference: Improved Canavanine-Derived Inhibitors of the Proprotein Convertase Furin
Source: ACS Med Chem Lett. 2021 Feb 9;12(3):426–32. doi: 10.1021/acsmedchemlett.0c00651 (PMC7957917; doi:10.1021/acsmedchemlett.0c00651)
Supplement: Supplementary file 1 — ml0c00651_si_002.pdf [file ml0c00651_si_002.pdf]

Supporting Information

The basicity makes the difference – Improved canavanine-derived inhibitors of the proprotein convertase furin

Thuy Van Lam van[1], Miriam Ruth Heindl[2], Christine Schlutt[1], Eva Böttcher-Friebertshäuser[2], Ralf Bartenschlager[3,4] Gerhard Klebe[1], Hans Brandstetter[5], Sven O. Dahms[1,5], Torsten Steinmetzer[1*]

[1]Institute of Pharmaceutical Chemistry, Philipps University, Marbacher Weg 6, D-35032 Marburg, Germany

[2]Institute of Virology, Philipps University, Hans-Meerwein-Str. 2, Marburg, Germany

[3]Department of Infectious Diseases, Molecular Virology, Heidelberg University, Im Neuenheimer Feld 344, 69120 Heidelberg, Germany

[4]German Center for Infection Research, Heidelberg Partner Site, Im Neuenheimer Feld 344, 69120 Heidelberg, Germany

[5]Department of Biosciences, University of Salzburg, Billrothstrasse 11, A-5020 Salzburg, Austria

Content

1.	Used analytical methods and synthesis	S3
2.	Analytical data of final inhibitors	S7
3.	Protein crystallography	S11
4.	Structure determination of human furin in complex with inhibitors **4**, **5**, **6**, and **8**	S12
5.	Structures of furin in complex with inhibitors **4**, **5**, **6**, and **8**	S13
6.	Enzyme kinetic measurements with furin and selected trypsin-like serine proteases	S14
7.	RSV infections and inhibition of multicycle replication	S17
8.	Inhibition of Dengue-2 virus and West Nile virus	S19
9.	Acute toxicity study in mice	S20
10.	Pharmacokinetic characterization of inhibitor **8** in rats	S21
11.	Abbreviations used in the supporting information	S23
12.	References	S23

1. Used analytical methods and synthesis

General

Protected Fmoc-amino acids, coupling reagents, and the used 2-chlorotritylchloride (2-CTC) resin for peptide synthesis were obtained from IRIS Biotech (Marktredwitz, Germany), ChemPur Feinchemikalien und Forschungsbedarf GmbH (Karlsruhe, Germany), and Bachem AG (Bubendorf, Switzerland). Solvents and standard reagents for synthesis were purchased from Sigma-Aldrich (Taufkirchen, Germany) and Thermo Fisher GmbH (Kandel, Germany), and used without further purification.

Analytical HPLC

Analytical HPLC experiments were performed on a Primaide (VWR, Hitachi) system (column, NUCLEODUR C18 ec, 5 µm, 100 Å, 4.6 mm × 250 mm, Macherey-Nagel, Düren, Germany). Water (solvent A) and acetonitrile (solvent B), both containing 0.1 % TFA, were used as eluents with a linear gradient (increase of 1 % solvent B/min) and a flow rate of 1 mL/min. The detection was performed at 220 nm.

Preparative HPLC

The final inhibitors were purified via preparative HPLC (pumps, Varian PrepStar model 218 gradient system; detector, ProStar model 320; fraction collector, Varian model 701) using a reversed phase column (NUCLEODUR C_{18} ec, 5 µm, 100 Å, 32 mm × 250 mm, Macherey-Nagel, Düren, Germany) with identical solvents as described for the analytical HPLC and a linear gradient at a flow rate of 20 mL/min (detection at 220 nm). The inhibitors were obtained as TFA salts after lyophilization on a freeze-drier (Martin Christ Gefriertrocknungsanlagen GmbH, Osterode am Harz, Germany). All final inhibitors possess a purity > 95 % based on HPLC-detection at 220 nm.

Mass spectrometry

The molecular mass of the synthesized compounds was determined using a QTrap 2000 ESI spectrometer (Applied Biosystems).

NMR spectroscopy

The ^{1}H NMR and ^{13}C NMR spectra for these inhibitors were defined by using a Jeol-ECX500 (Jeol Inc., Peabody, MA).

Synthesis

The synthesis of the inhibitors was performed as described below in detail for compound **8** (Scheme S1).

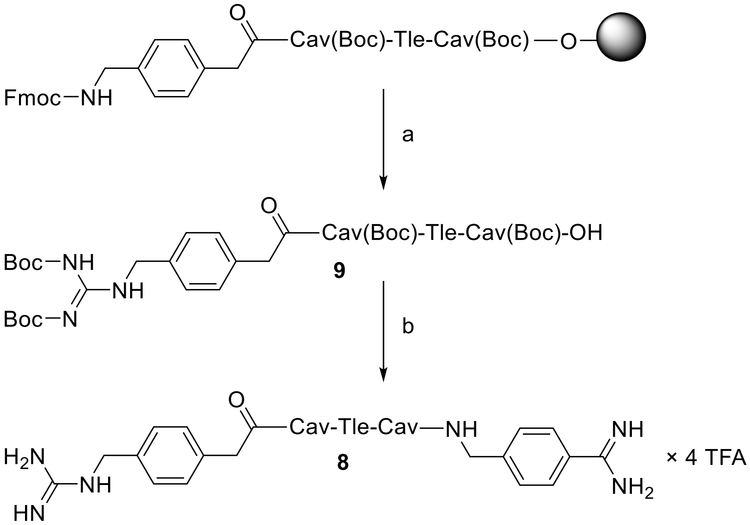

Scheme S1. Synthesis of inhibitor **8** on 2-CTC resin, loaded with Fmoc-Cav(Boc)-OH. The following P3, P4, and P5 residues were coupled by a standard Fmoc-SPPS protocol using a 2.0-fold excess of Fmoc amino acids, HBTU, and HOBt in presence of 4 equiv DIPEA. The Fmoc deprotection was always performed with 20 % piperidine in DMF. Reagents and conditions: (a) (i) 20 % piperidine in DMF, (ii) 3.0 equiv of N,N'-Bis-Boc-1-guanylpyrazole, 4.0 equiv. of DIPEA, DMF, overnight, (iii) 1 % TFA in DCM, 3 × 30 min; neutralization by DIPEA (b) (i) 1.0 equiv. of 4-amidinobenzylamide · 2 HCl, HBTU, and 3 equiv. of DIPEA and 6-Cl-HOBt; (ii) TFA/TIS/H2O (95:2.5:2.5 v/v/v), 2 h at rt, precipitation in diethyl ether, followed by preparative RP-HPLC.

The side chain protected P5-P2 segment of inhibitor **8** was prepared by solid phase peptide synthesis (SPPS) on 2-chlorotritylchloride (2-CTC) resin, as described previously.[1] In brief, 500 mg of the 2-CTC-resin (loading 1.6 mmol/g, absolute 0.8 mmol) was treated with 399 mg (0.8 mmol) Fmoc-Cav(Boc)-OH dissolved in 8 mL dry DCM and 0.557 ml (3.2 mmol, 4 equiv) diisopropylethylamine (DIPEA) in a 10 mL polypropylene syringe with filter frit (Multisyntech GmbH, Witten, Germany). The resin was shaken for 2 hours, and subsequently washed (3 × 1 min) with DCM/MeOH/DIPEA (17:2:1, v/v/v), 3 × with DCM, 3 × with DMF, 4 × with DCM. Afterwards, the resin was dried *in vacuo* providing 720 mg Fmoc-Cav(Boc)-resin (loading 0.661 mmol/g).

100 mg of this Fmoc-Cav(Boc)-resin (loading 0.661 mmol/g, absolute 0.0661 mmol) was used for the following SPPS steps using a standard Fmoc protocol performed in a 2 mL polypropylene syringe with filter frit. The Fmoc group was always removed by treatment with 20 % piperidine in DMF (5 and 15 min), followed by subsequent washing with DMF (7 × 1 min). For the coupling of the following residues (Fmoc-Tle-OH, Fmoc-Cav(Boc)-OH, and Fmoc-4-aminomethyl-phenylacetic acid) a 2.0-fold excess of Fmoc amino acids, HBTU, and HOBt in presence of 4 equiv DIPEA was used. When using a higher excess of Fmoc amino acids, a considerable amount of side product was observed due to an additional acylation on the Boc protected Cav side chain. This indicated that the Fmoc-Cav(Boc)-OH is an acceptable building block for the synthesis of short peptide segments, but is not perfectly suited for longer sequences, when using standard SPPS conditions with 3- or 4-fold excess of Fmoc-amino acids and coupling reagent.

After the final coupling of Fmoc-4-aminomethyl-phenylacetic acid, washing with DMF (4 × 1 min), Fmoc deprotection, and subsequent washing with DMF (5 × 1 min), the resin was treated with 62 mg (0.198 mmol, 3.0 equiv) of N,N'-Bis-Boc-1-guanylpyrazole[2] and 46 µl (0.264 mmol, 4.0 equiv) of DIPEA dissolved in 1.5 mL DMF for the conversion of the N-terminal aminomethyl substituent into a bis-Boc-protected guanidinomethyl group. The resin was shaken overnight and subsequently washed with DMF (3 × 1 min) and DCM (5 × 1 min). The protected intermediate **9** (Scheme S1) was removed from the resin under mild acidic conditions with 1.5 ml of 1 % TFA in DCM (3 × 30 min). After each cleavage step, the solution was immediately neutralized by treatment with DIPEA. The solvent was removed in vacuo providing an oily residue (HPLC retention time 23.47 min, start at 30 % solvent B; MS calcd m/z 1036.56, found m/z 1037.56, $(M+H)^+$).

The crude intermediate **9** was treated with 22 mg (0.0992 mmol, 1.5 equiv based on the initial resin loading) 4-amidinobenzylamide · 2 HCl,[3] 52 mg (0.0992 mmol, 1.5 equiv) PyBOP, 50.5 mg (0.298 mmol, 4.5 equiv) 6-Cl-HOBT, and 43 µL (0.248 mmol, 3.75 equiv) DIPEA dissolved in 2 mL DMF. The mixture was stirred at room temperature overnight, afterwards the solvent was removed in vacuo. The remaining oily residue (HPLC retention time 20.12 min, start at 30 % solvent B) was deprotected under strong acidic conditions using 3 mL TFA/H_20/triisopropylsilane (95:2.5:2.5, v/v/v) for 2 hours. The solution was subsequently dropwise added to cold diethyl ether. The precipitated crude peptide obtained after centrifugation was purified by preparative reversed-phase HPLC, providing 22.3 mg (0.018 mmol, 27.5 % based on the used 100 mg Fmoc-Cav(Boc)-resin) of the final inhibitor **8** as TFA

salt after lyophilization (purity > 95 % based on HPLC-detection at 220 nm, HPLC retention time 21.4 min, start at 1 % solvent B; MS calcd m/z 767.43, found m/z 768.40, $(M+H)^+$).

For all other inhibitors, very similar yields between 19-24 mg were obtained, when starting from 100 mg of the loaded Fmoc-Cav(Boc)-resin (for inhibitors **3**, **4**, and **7**) or Fmoc-Lys(Boc)-resin (for compounds **5** and **6**).

2. Analytical data of final inhibitors

Table S1. Analytical data of the newly synthesized inhibitors **3-8**.

No.	Chemical formula	HPLC (min)[a]	MS calc.	MS found $[M+H]^+$
**3**	$C_{34}H_{53}N_{13}O_5$	20.0	723.43	724.45
**4**	$C_{35}H_{55}N_{15}O_5$	21.1	765.45	766.37
**5**	$C_{34}H_{53}N_{11}O_5$	17.0	695.42	696.44
**6**	$C_{35}H_{55}N_{13}O_5$	20.2	737.44	738.30
**7**	$C_{33}H_{51}N_{13}O_6$	20.3	725.41	726.40
**8**	$C_{34}H_{53}N_{15}O_6$	21.4	767.43	768.40

[a]start at 1 % solvent A

NMR spectroscopy

The ^{1}H NMR spectra for inhibitors **3** – **8** and the ^{13}C NMR spectra for the most potent furin inhibitors **6** and **8** were recorded by using a Jeol-ECX500 (Jeol Inc., Peabody, MA). Samples were dissolved in DMSO-*d6* and the solvent residual signal was used as reference (^{1}H-NMR: 2.50 ppm, ^{13}C-NMR: 39.52 ppm).[4] Chemical shifts δ are given in the unit ppm, coupling constants J in the unit Hz.

Inhibitor **3**

^{1}H NMR (500 MHz, [D$_6$] DMSO) δ = 11.21 (s, 1H), 9.19 (d, *J* = 60.0 Hz, 4H), 8.64 (t, *J* = 6.1 Hz, 1H), 8.42 (d, *J* = 8.0 Hz, 1H), 8.31 – 8.05 (m, 4H), 7.89 – 7.63 (m, 7H), 7.57 (d, *J* = 9.0 Hz, 1H), 7.51 – 7.22 (m, 10H), 4.51 – 4.31 (m, 4H), 4.24 (d, *J* = 9.0 Hz, 1H), 3.99 (s, 2H), 3.82 (t, *J* = 6.5 Hz, 2H), 3.59 – 3.43 (m, 2H), 3.08 (dt, *J* = 12.7, 6.6 Hz, 2H), 2.06 (dq, *J* = 14.1, 7.3 Hz, 1H), 1.97 – 1.85 (m, 1H), 1.76 – 1.63 (m, 1H), 1.59 – 1.34 (m, 3H), 0.88 ppm (s, 9H).

Inhibitor **4**

^{1}H NMR (500 MHz, [D$_6$] DMSO) δ = 11.22 (s, 1H), 9.26 (s, 2H), 9.14 (s, 2H), 8.65 (t, *J* = 6.1 Hz, 1H), 8.42 (d, *J* = 8.0 Hz, 1H), 8.25 (d, *J* = 7.5 Hz, 1H), 8.03 (t, *J* = 6.2 Hz, 1H), 7.84 – 7.63 (m, 7H), 7.56 (d, *J* = 9.0 Hz, 1H), 7.50 – 7.38 (m, 3H), 7.23 (h, *J* = 9.2 Hz, 11H), 4.48 – 4.19 (m, 7H), 3.82 (t, *J* = 6.3 Hz, 2H), 3.57 – 3.40 (m, 2H), 3.09 (dq, *J* = 12.1, 6.7, 6.1 Hz, 2H), 2.12 – 2.00 (m, 1H), 1.96 – 1.83 (m, 1H), 1.75 – 1.63 (m, 1H), 1.60 – 1.38 (m, 3H), 0.87 ppm (s, 9H).

Inhibitor **5**

^{1}H NMR (500 MHz, [D$_6$] DMSO): δ = 11.26 (s, 1H), 9.25 (d, *J* = 12.9 Hz, 4H), 8.57 (t, *J* = 6.0 Hz, 1H), 8.49 (d, *J* = 8.1 Hz, 1H), 8.21 (s, 3H), 8.11 (d, *J* = 7.5 Hz, 1H), 7.90 – 7.73 (m, 9H), 7.65 (d, *J* = 9.3 Hz, 1H), 7.45 (d, *J* = 8.5 Hz, 2H), 7.36 (d, *J* = 8.3 Hz, 2H), 7.28 (d, *J* = 8.3 Hz, 2H), 4.59 – 4.39 (m, 2H),

4.35 – 4.18 (m, 3H), 4.00 (s, 2H), 3.80 (t, *J* = 6.7 Hz, 2H), 3.60 – 3.45 (m, 2H), 2.75 (s, 2H), 2.11 – 1.96 (m, 1H), 1.87 (dt, *J* = 14.3, 7.4 Hz, 1H), 1.73 – 1.47 (m, 4H), 1.41 – 1.20 (m, 2H), 0.87 ppm (s, 9H).

Inhibitor **6**

^{1}H NMR (500 MHz, [D$_6$] DMSO) δ = 11.31 (s, 1H), 9.31 (d, *J* = 32.6 Hz, 4H), 8.57 (t, *J* = 6.0 Hz, 1H), 8.48 (d, *J* = 8.0 Hz, 1H), 8.16 (t, *J* = 6.1 Hz, 1H), 8.11 (d, *J* = 7.5 Hz, 1H), 7.83 (s, 7H), 7.76 (d, *J* = 8.5 Hz, 2H), 7.63 (d, *J* = 9.1 Hz, 1H), 7.45 (d, *J* = 8.5 Hz, 2H), 7.23 (q, *J* = 8.3 Hz, 8H), 4.54 – 4.18 (m, 7H), 3.80 (t, *J* = 6.6 Hz, 2H), 3.63 – 3.43 (m, 2H), 2.74 (s, 2H), 2.04 (m, 1H), 1.87 (dd, *J* = 14.3, 8.5 Hz, 1H), 1.76 – 1.47 (m, 4H), 1.40 – 1.19 (m, 2H), 0.86 ppm (s, 9H).

^{13}C NMR (126 MHz, [D$_6$] DMSO) δ = 171.55, 170.77, 170.53, 169.70, 165.44, 158.45, 156.97, 145.77, 135.33, 135.28, 129.18, 127.94, 127.25, 127.10, 126.43, 73.20, 59.63, 52.69, 49.65, 43.69, 41.71, 41.52, 38.62, 34.29, 31.08, 29.83, 26.64, 26.48, 22.35 ppm.

Inhibitor **7**

^{1}H NMR (500 MHz, [D$_6$] DMSO) δ = 11.25 (d, *J* = 29.2 Hz, 2H), 9.25 (d, *J* = 17.4 Hz, 4H), 8.64 (s, 1H), 8.50 (d, *J* = 8.1 Hz, 1H), 8.33 – 8.13 (m, 4H), 7.82 – 7.72 (m, 10H), 7.66 (d, *J* = 9.0 Hz, 1H), 7.46 (d, *J* = 8.5 Hz, 2H), 7.36 (d, *J* = 8.3 Hz, 2H), 7.28 (d, *J* = 8.2 Hz, 2H), 4.51 (td, *J* = 8.6, 5.3 Hz, 1H), 4.46 – 4.32 (m, 3H), 4.25 (d, *J* = 9.0 Hz, 1H), 4.04 – 3.96 (m, 2H), 3.81 (dt, *J* = 19.1, 6.8 Hz, 3H), 3.58 – 3.47 (m, 3H), 2.04 (tt, *J* = 13.5, 7.3 Hz, 2H), 1.96 – 1.80 (m, 2H), 0.88 ppm (s, 9H).

Inhibitor **8**

^{1}H NMR (500 MHz, [D$_6$] DMSO) δ = 11.36 (s, 1H), 11.28 (s, 1H), 9.33 (s, 2H), 9.27 (s, 2H), 8.65 (t, *J* = 6.1 Hz, 1H), 8.50 (d, *J* = 8.1 Hz, 1H), 8.27 (d, *J* = 7.4 Hz, 1H), 8.14 (t, *J* = 6.1 Hz, 1H), 7.83 (s, 8H), 7.76 (d, *J* = 8.5 Hz, 2H), 7.64 (d, *J* = 9.0 Hz, 1H), 7.46 (d, *J* = 8.5 Hz, 4H), 7.29 – 7.18 (m, 6H), 4.54 – 4.30 (m, 6H), 4.24 (d, *J* = 9.0 Hz, 1H), 3.82 (dt, *J* = 17.7, 6.6 Hz, 4H), 3.50 (d, *J* = 2.1 Hz, 2H), 2.11 – 2.00 (m, 2H), 1.95 – 1.82 (m, 2H), 0.87 ppm (s, 9H).

^{13}C NMR (126 MHz, [D$_6$] DMSO) δ = 171.29, 170.81, 170.60, 169.98, 165.44, 158.50, 156.97, 145.53, 135.33, 135.27, 129.18, 127.97, 127.24, 127.10, 126.50, 73.22, 72.93, 59.69, 49.78, 49.56, 43.70, 41.78, 41.52, 39.85, 34.25, 29.98, 29.78, 26.46 ppm.

As examples, the ^{1}H and ^{13}C NMR spectra of inhibitors **6** (Figure S1) and **8** (Figure S2) are shown below. The structures of the inhibitors are provided in their fourfold protonated form obtained after preparative HPLC under acidic conditions (pH approximately 1.9). The provided chemical formulas below the structures are calculated without the trifluoroacetate counterions.

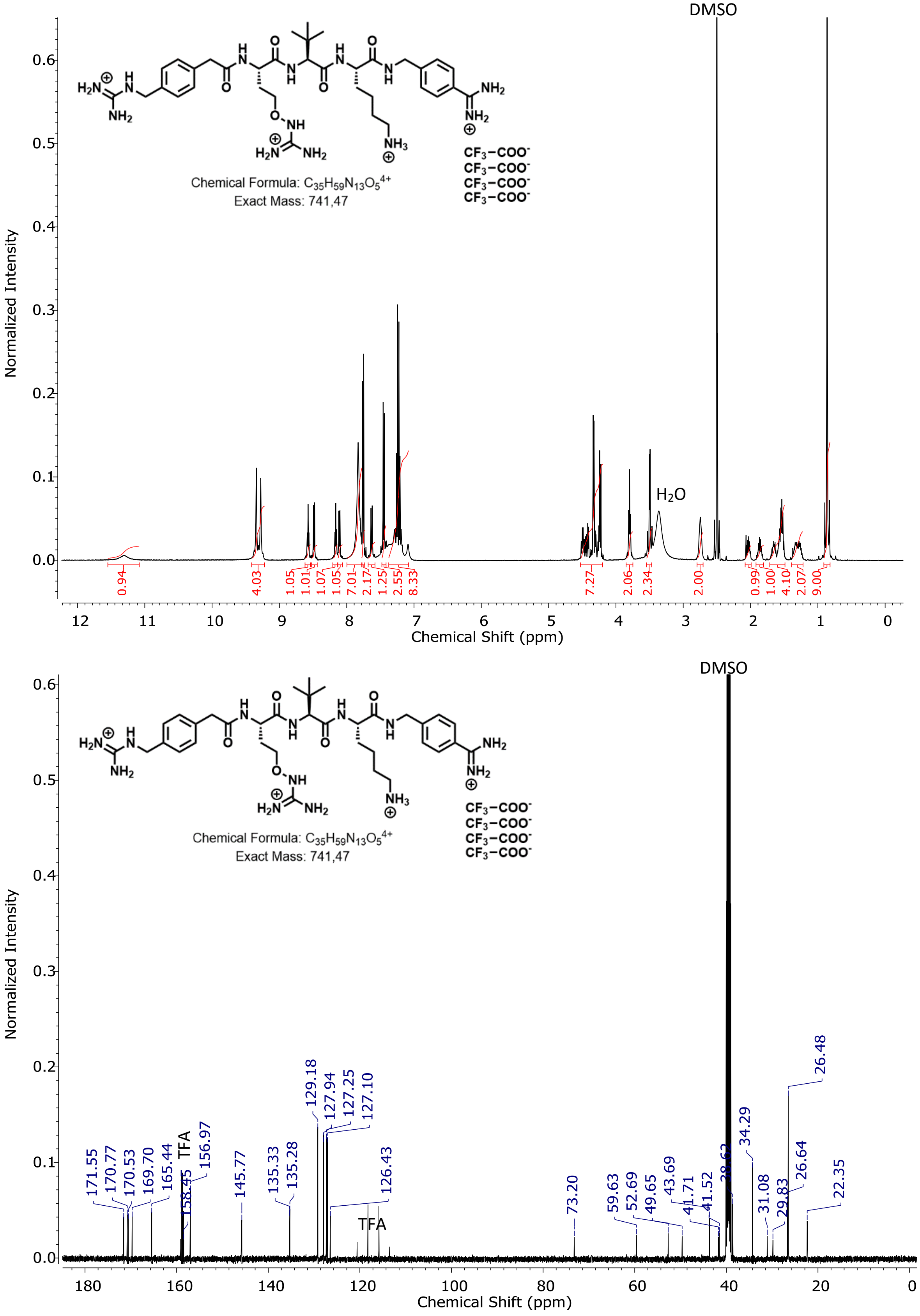

Figure S1. ^{1}H- and ^{13}C-NMR spectra of inhibitor **6** · 4 TFA in DMSO-*d6*.

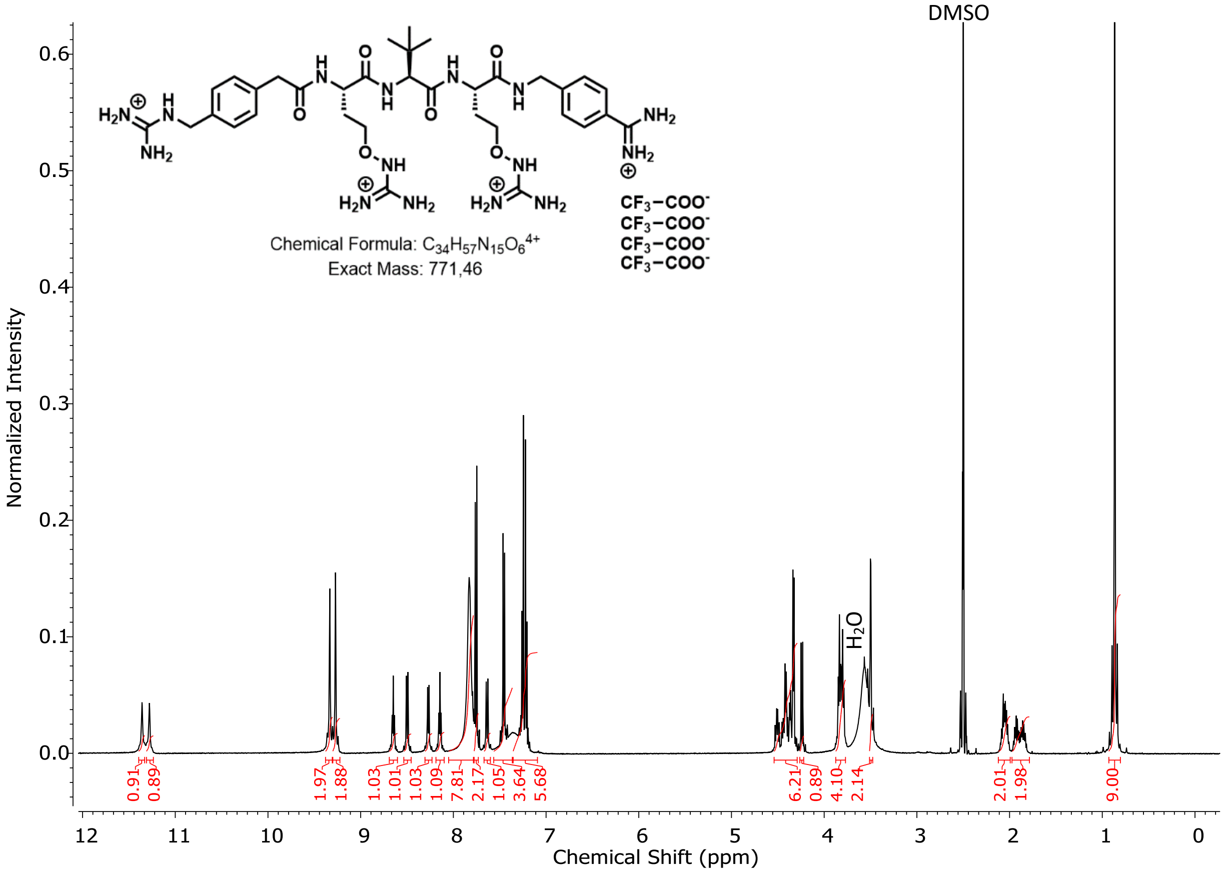

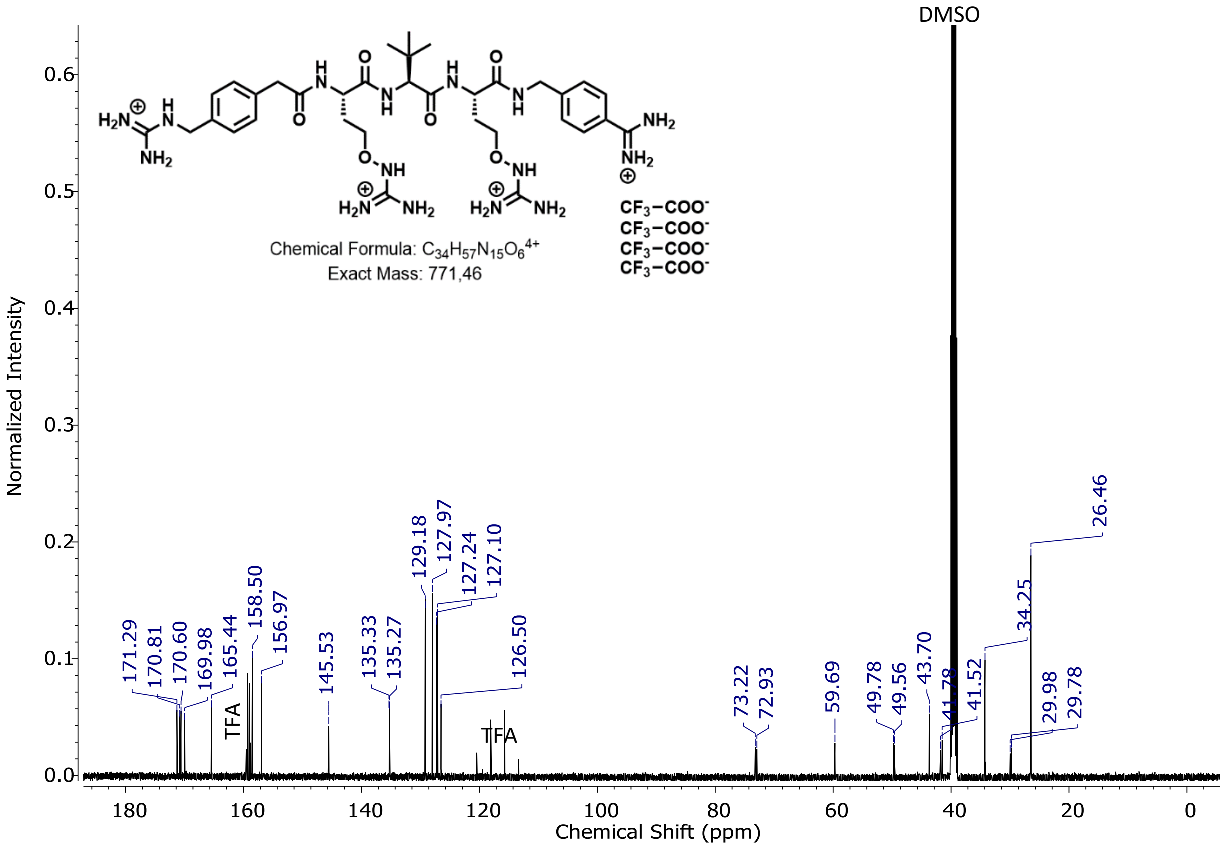

Figure S2. ^{1}H- and ^{13}C-NMR spectra of inhibitor **8** · 4 TFA in DMSO-*d6*.

3. Protein crystallography

Equal volumes of homogeneously glycosylated human furin,[5] ~9 mg/mL in 10 mM Hepes pH 7.5, 100 mM NaCl, 2 mM $CaCl_2$ and crystallization solution (100 mM MES, 200 mM K/NaH_2PO_4, pH 5.5-6.0 and 2 M NaCl) were mixed and equilibrated against the reservoir (3.0-3.6 M NaCl) in vapor diffusion experiments at 18-20°C as described previously.[6-7]

The soaking solution (3.13 M NaCl; 100 mM Mes/NaOH; pH 5.5; 200 mM NaH_2PO_4; 1 mM $CaCl_2$; 20% DMSO) was supplemented with 5 mM of inhibitors **5**, **6**, **7** or **8**. Crystals were soaked for ~16 h and flash cooled in liquid N_2. Diffraction data were collected at the synchrotron beam line MASSIF-3 (ID30A-3[8]) of the European Synchrotron Radiation Facility (ESRF) and at BL14.2[9] (BESSY-II) of the Helmholtz-Zentrum Berlin (HZB). The data were processed using XDS[10] (v.03/2019) with XDS-APP[11] (v2.0) and the CCP4 program suite[12] (v.7.0.078). COOT[13] (v.0.8.9.2) was used for model building. Refinement was performed in PHENIX[14] (v.1.17.1) using the PDB-ID 6EQX[6] as initial model. One specific R_{free}-set (initially generated up to 1.0 Å[7]) was transferred to the data sets prior refinement start. Geometry restraints of the inhibitors were obtained from the PRODRG-server.[15] Electron density omit maps were calculated in PHENIX[14] (v.1.17.1). PYMOL was used for molecular graphics (http://www.pymol.org). The coordinates and structure factors of furin in complex with **5**, **6**, **7** and **8** have been deposited at the World Wide Protein Data Bank with the PDB IDs 6YD2, 6YD3, 6YD4 and 6YD7, respectively.

4. Structure determination of human furin in complex with inhibitors 4, 5, 6, and 8

Table S2. Data collection and refinement statistics.

	**4**	**5**	**6**	**8**
**Data collection statistics**				
PDB ID	6YD7	6YD2	6YD3	6YD4
Wavelength	0.9184	0.9677	0.9677	0.9184
Space group	$P6_522$	$P6_522$	$P6_522$	$P6_522$
Unit cell parameters: a = b, c	131.8, 155.4	131.5, 155.2	131.6, 155.3	131.7, 155.5
Resolution range [a] (Å)	47.2-1.8 (1.91-1.80)	43.1-1.8 (1.91-1.80)	43.1-2.0 (2.12-2.00)	47.2-1.7 (1.80-1.70)
R_{meas} [a] (%)	14.1 (160.9)	13.7 (203.9)	21.4 (139.8)	11.7 (197.0)
I/sigI [a]	21.24 (2.4)	19.2 (1.8)	12.1 (2.1)	22.21 (2.0)
Completeness [a]	98.6 (97.6)	98.4 (98.3)	97.9 (98.6)	99.9 (99.5)
No. of observations	1473091 / 73093	1,244,968 / 72,501	905,715 / 52,910	1754091 / 87466
**Refinement statistics**				
No. of non-hydrogen atoms	4,211	4,179	4,159	4,237
protein/inhibitor/other	3670 / 55 / 465	3,653 / 61 / 444	3,653 / 67 / 418	3,670 / 69 / 477
R_{work}/R_{free}	15.4 / 17.1	15.8 / 17.7	16.0 / 18.5	15.3 / 16.9
B-factors (Å^2)				
Overall/Wilson plot	29.7 / 30.4	32.1 / 32.4	32.0 / 31.3	31.2 / 31.3
protein/inhibitor/other	28.6 / 25.8 / 38.1	31.1 / 27.3 / 40.3	31.4 / 25.7 / 38.4	30.1 / 26.8 / 40.2
RMSD bond length (Å)	0.009	0.010	0.011	0.009
RMSD bonded B-factors	4.4	4.5	4.8	4.3

[a] Values of the highest resolution shell are given in parentheses

5. Structures of furin in complex with inhibitors 4, 5, 6, and 8

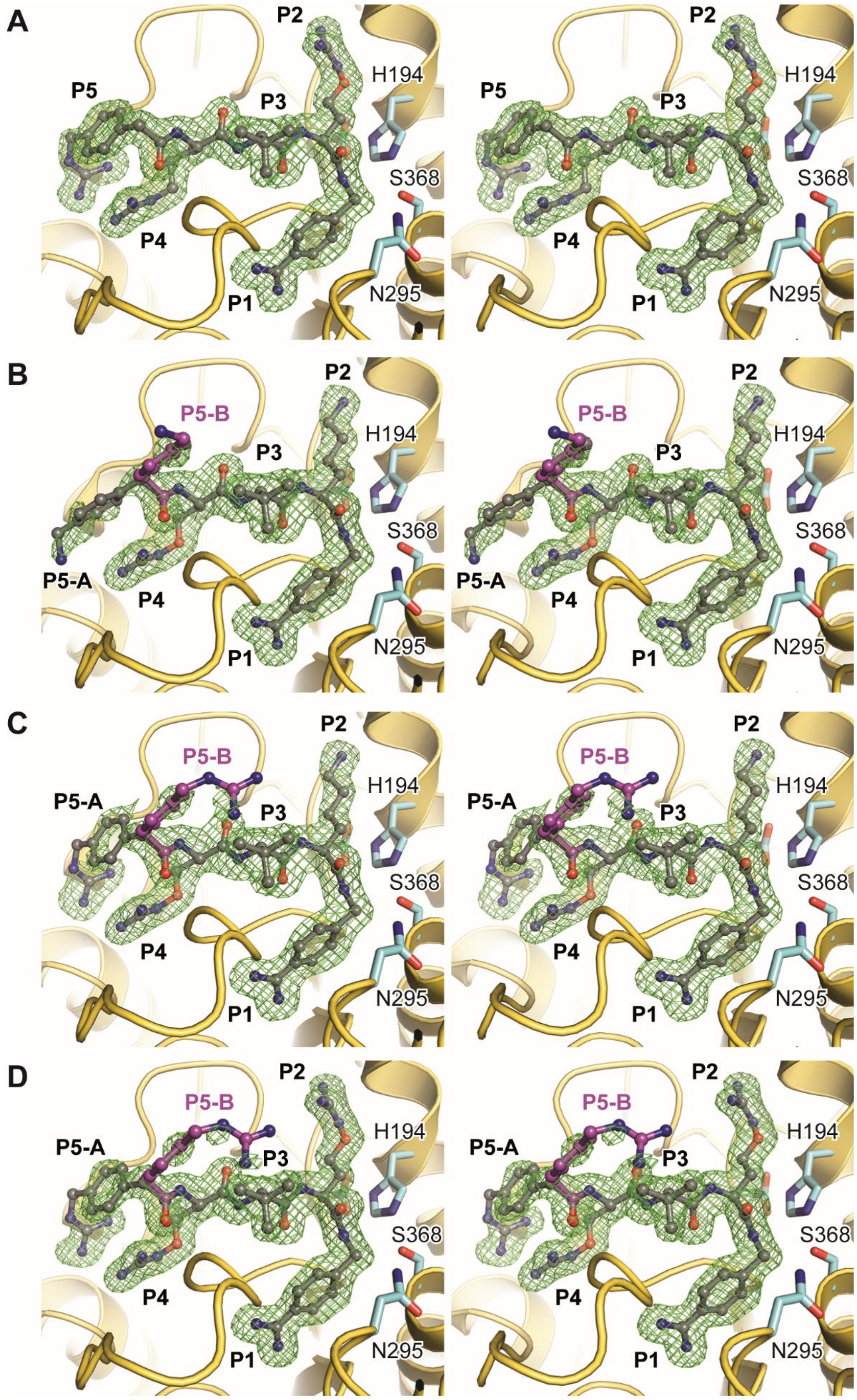

Figure S3. Stereo representation of the difference electron density maps of the furin-bound inhibitors **4** (A), **5** (B), **6** (C), and **8** (D). The protein backbone of furin is given as cartoon representation (golden). The residues Ser368 and His194 of the catalytic triad as well as Asn295 of the oxyanion hole are shown as stick model with carbon atoms in cyan. Inhibitors are shown as ball-and-stick models. The $\mathbf{F_o}$-$\mathbf{F_c}$ simulated annealing omit electron density maps (green mesh, atoms of the inhibitors were deleted for map calculation to avoid model bias) are contoured at 3σ. The alternative conformations of the P5 residue in the complexes shown in panels B-D are colored in magenta.

6. Enzyme kinetic measurements with furin and selected trypsin-like serine proteases

Measurements with furin

All measurements were performed in black 96-well plates (Nunc, Langenselbold, Germany) at room temperature with a microplate reader Safire 2 (Tecan, Switzerland) at λ_{ex} 380 nm and λ_{em} 460 nm as described previously.[16] Each well contained 2 µL inhibitor solution (dissolved in DMSO), 20 µL of Phac-Arg-Val-Arg-Arg-AMC as substrate (dissolved in water, 12.5 µM in assay) and 160 µL buffer (100 mM HEPES, 0.2 % Triton X-100, 2 mM $CaCl_2$, 0.02 % sodium azide und 1 mg/mL BSA, pH 7.0). The measurements were started by addition of 20 µL furin[17] solution (0.95 nM in assay). Using the slopes of the linear progress curves the rates were calculated, which were fitted as function of the inhibitor concentration using equation (**1**) for tight-binding inhibitors,[18] whereby v_0 is the velocity in absence of an inhibitor, I_t is the total inhibitor concentration, E_t is the total enzyme concentration, and K_i^* is the apparent inhibition constant at the used substrate concentration.

$$v = v_0 \times \frac{\left[(K_i^* + I_t - E_t)^2 + 4 \times K_i^* \times E_t\right]^{1/2} - (K_i^* + I_t - E_t)}{2 \times E_t} \qquad (\mathbf{1})$$

The apparent K_i^* was converted into the true K_i using equation (**2**), whereby S is the constant concentration (12.5 µM in assay) of the AMC-substrate Phac-Arg-Val-Arg-Arg-AMC and K_m describes the value of Michaelis constant.

$$K_i = \frac{K_i^*}{1 + \frac{S}{K_m}} \qquad (\mathbf{2})$$

The provided K_i values are the average ± standard deviation of at least three measurements.

Measurements with selected trypsin-like serine proteases

For selectivity studies, the inhibitors were also tested against four trypsin-like serine proteases including trypsin, the clotting proteases thrombin and factor Xa, and the fibrinolytic plasmin (Table S3). The

reference inhibitors **1** and **2** still possess a relatively potent inhibition of trypsin with K_i values of 52 nM and 100 nM, whereas both compounds are only poor inhibitors of the other three tested proteases (K_i values > 3 µM). Notably, a more than 10 fold reduced trypsin inhibition was found for the canavanine-derived inhibitors **5**-**8**, they also possess a negligible inhibitory potency against thrombin, factor Xa, and plasmin.

Table S3. Selectivity measurements with trypsin-like serine proteases, the K_i values for furin are provided as reference. Only for trypsin, an additional selectivity ratio (SR) compared to the furin inhibition is provided in brackets below the K_i value. For the other tested proteases the SR is further increased (not calculated).

		K_i			
**No.**	**furin (pM)**	**trypsin (µM)**<br>**SR ($K_{i\ trypsin}/K_{i\ furin}$)**	**thrombin (µM)**	**factor Xa (µM)**	**plasmin (µM)**
**1**	5.5 ± 0.3	0.052 ± 0.0049<br>(9454)	20 ± 3.2	7.1 ± 0.37	3.5 ± 0.33
**2**	8.8 ± 4.9	0.10 ± 0.018<br>(11364)	44 ± 17	8.0 ± 0.57	8.8 ± 0.62
**3**	76.8 ± 14.9	0.21 ± 0.002<br>(2734)	13 ± 0.09	28 ± 0.37	3.3 ±0.25
**4**	13.1 ± 1.6	0.22 ± 0.2<br>(16794)	9.6 ± 0.18	15 ± 3.5	3.1 ± 0.26
**5**	114 ± 25.9	1.3 ± 0.049<br>(11403)	10 ±0.06	18 ± 0.60	21 ± 0.87
**6**	36.3 ± 11.2	0.55 ± 0.012<br>(15152)	61 ± 3.4	26 ± 0.41	28 ± 0.44
**7**	34.2 ± 5.1	1.3 ± 0.1<br>(38012)	25 ± 0.59	47± 2.1	19 ± 0.88
**8**	10.1 ± 3.2	0.69 ± 0.001<br>(68317)	22 ±0.52	36 ± 3.5	12 ± 0.21

The measurements with trypsin, thrombin, factor Xa, and plasmin were performed at room temperature in 50 mM Tris × HCl buffer pH 8.0 containing 154 mM NaCl, as described previously[19]. The assays were conducted in black 96 well plates using a Fluoroskan Ascent® plate reader (Thermo Fisher Scientific, Vantaa, Finland) with λ_{ex} 355 and λ_{em} 460 nm. The measurements were performed with 100 µL buffer containing the inhibitor, 20 µL substrate dissolved in water and were started by addition of 20 µL enzyme solution (total assay volume 140 µL). Mes-DArg-Pro-Arg-AMC[20] was used as fluorescence substrate for human fXa (97 pM in assay, molecular weight 46 kDa, Enzyme Research South Bend, Indiana, USA). Tos-Gly-Pro-Arg-AMC was used for bovine thrombin prepared according to Walsmann[21] (31 pM in assay, molecular weight 33.6 kDa), and Mes-DArg-Gly-Arg-AMC[20] for porcine trypsin (30 pM in assay, molecular weight 23.2 kDa, Merck, Darmstadt, Germany). Assays with human plasmin (molecular weight 78 kDa, Chromogenix, Lexington, USA) were performed with an enzyme concentration of 0.35 nM in the assay using the substrate Mes-DArg-Phe-Arg-AMC[19]. The provided K_i values are the average ± standard deviation of three measurements.

7. RSV infections and inhibition of multicycle replication

The influence of the inhibitors on the viability of the used A549 cells was determined by using the CellTiter-Glo® assay (Promega GmbH, Mannheim, Germany) according to the instructions of the manufacturer. No cytotoxicity was observed at an inhibitor concentration of 50 µM (Figure S4).

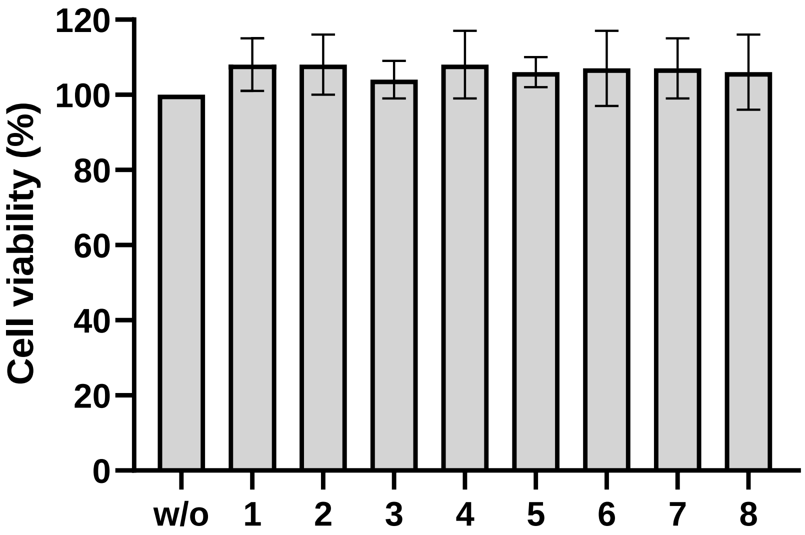

Figure S4. Cytotoxicity of the used inhibitors at a concentration of 50 µM after 24 hours incubation with A549 cells. The test was performed by the CellTiter-Glo® assay.

The experiments were conducted as recently described[22]. RSV A2 strain was kindly provided by Christine Krempl, Institute of Virology and Immunobiology, University of Würzburg, Würzburg, Germany. Cell growth and incubations occurred at 37 °C and 5 % CO_2. All infection experiments were performed in minimal essential medium (MEM, Gibco, Thermo Fisher Scientific, Paisley, UK) supplemented with 5 % fetal calf serum (FCS), penicillin, streptomycin, and glutamine. RSV was propagated in Hep-2 cells. Concentrated virus stocks were prepared from both cell supernatant and infected cells as described previously.[22] Briefly, infected cells were mechanically disrupted by vortexing to release cell-associated virus. The cell suspension and the virus-containing Hep-2 cell culture supernatant were cleared by low-speed centrifugation, subsequently combined, supplemented with 0.1 M $MgSO_4$ and 0.05 M HEPES and stored as virus stock at -80°C. Multicycle virus replication in the presence of inhibitors was analyzed in A549 human lung cancer cells. A549 cells were infected with RSV at a multiplicity of infection (MOI) of 1 for 1 h. The inoculum was then removed and cells were incubated in fresh medium with or without inhibitors for 72 h. At 16, 24, 48 and 72 h post infection virus

progeny released into the cell supernatants were quantified by plaque assay using Vero76 cells with Avicel overlay.[23] Briefly, cells were incubated with 10-fold serial dilutions of the supernatants in MEM supplemented with 5 % FCS, antibiotics and glutamine for 1 h, washed with PBS and incubated with Avicel overlay for 72 h. Cells were fixed and permeabilized and immunostained using a polyclonal goat antibody against RSV (BIO-RAD Laboratories GmbH, Feldkirchen, Germany), peroxidase-conjugated secondary antibodies and the peroxidase substrate TrueBlue (SeraCare, Milford, USA).

8. Inhibition of Dengue-2 virus and West Nile virus

The influence of the inhibitors on the viability of the used Huh-7 cells was determined by using the CellTiter-Glo® assay (Promega GmbH, Mannheim, Germany) according to the instructions of the manufacturer, as described previously.[24] A negligible cytotoxicity was observed at the used inhibitor concentrations up to 50 µM (Figure S5).

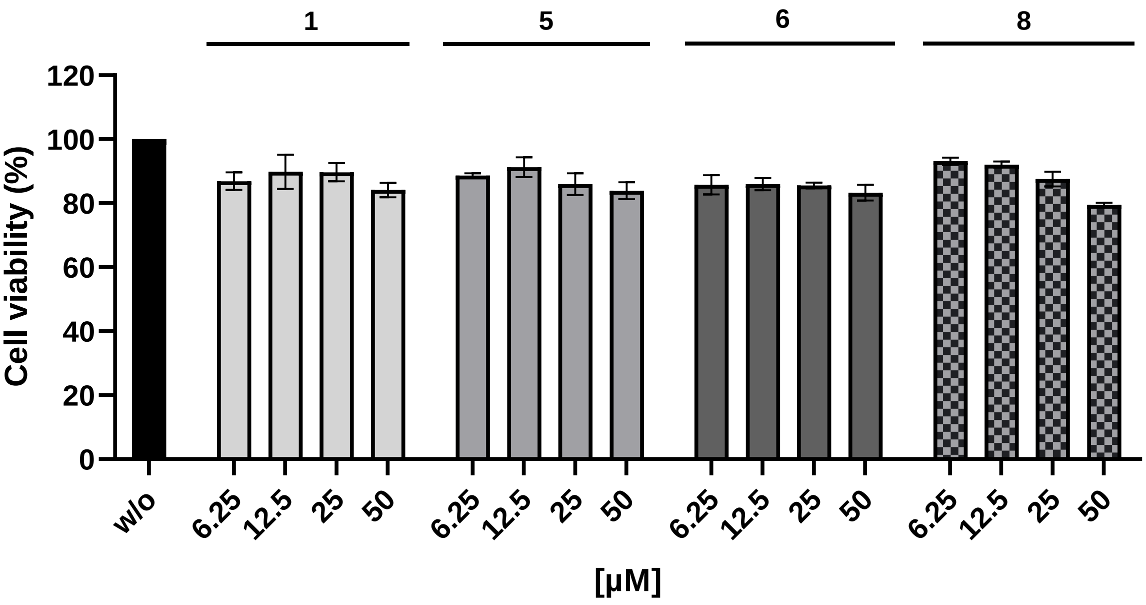

Figure S5. Cytotoxicity of the used inhibitors at the indicated concentrations after 48 hours incubation with Huh-7 cells. The test was performed by the CellTiter-Glo® assay.

Antiviral activity of furin inhibitors against Dengue-2 virus (DENV-2) and West Nile virus (WNV) has been described recently.[1, 25] In brief, DENV-2 replication and spread was determined by plaque assay using Huh-7 cells seeded into 96-well plates.[26-27] One day after seeding, cells were infected with DENV-2 (strain 16681) at a MOI of 1 PFU/cell. Compounds specified in the results section were dissolved in culture medium and added at different concentrations to the cells together with the virus. After a 2 h incubation at 37°C, media were replaced by fresh medium containing given concentrations of the inhibitors. Virus progeny contained in culture supernatants were quantified 48 h post infection by plaque assay on VeroE6 cells. Ribavirin was included as a reference compound. The analogous plaque assay was used in the case of WNV (strain New York 99; MOI = 0.2 PFU/cell). The impact of compounds on cell viability was determined by using CellTiter-Glo® and CytoTox 96® assays (Promega GmbH, Mannheim, Germany) following the instructions of the manufacturer.

9. Acute toxicity study in mice

The study was conducted by Eurofins Panlabs Taiwan Ltd. in ICR mice weighing 23 ± 3 g (provided by BioLasco Taiwan under Charles River Laboratories Licensee) according to a standard protocol as described previously.[1] To avoid TFA-related side effects, inhibitors were converted into physiologically more acceptable HCl salts as described previously.[1] All doses were calculated as the free bases of the inhibitors, dissolved in saline, and administered intraperitoneally (*ip.*) at 5 mL/kg to groups of four mice each (two male and two female). The animals received a certain initial dose of each inhibitor (2.5 mg/kg). If the whole group of animals survived for 24 hours, the dose for the same animals was increased (5, 10, and 15 mg/kg). All aspects of this work including housing, experimentation, and animal disposal were performed in general accordance with the "Guide for the Care and Use of Laboratory Animals: Eighth Edition" (National Academies Press, Washington, D.C., 2011) in an AAALAC-accredited laboratory animal facility. In addition, the animal care and use protocol was reviewed and approved by the IACUC at Eurofins Panlabs Taiwan, Ltd.

10. Pharmacokinetic characterization of inhibitor 8 in rats

The study was performed by Pharmacelsus GmbH Germany in adult male Sprague Dawley rats. The animals were housed in a temperature-controlled room (20-24°C) and maintained in a 12 h light/12 h dark cycle. Food and water were available throughout the duration of the study. As described before in the toxicity study, the HCl salt of compound **8** was used for the PK study. Test item formulations were administered either intravenously (*iv.*) at 1 mg/kg to a group of 3 rats or intraperitoneally (*ip.*) at 2.5 mg/kg to 2 rats, respectively. After treatment of all rats, six serial blood samples were obtained from each rat at 5 min, 15 min, 30 min, 1 h, 3 h, and 8 h. At these time points, a volume of 100 µL blood was obtained from the vascular access harness in tubes containing Li-Heparin. After sampling, the plasma was prepared within 10 min and was kept at -20°C until processed for bioanalysis. Both Liquid chromatography (Accela U-HPLC pump, Accela Open Autosampler (Thermo Fisher Scientific, USA)) and mass spectrometry (Q-Exactive mass spectrometer (Orbitrap™ technology with accurate mass)) were used for analysis. All experimental procedures were approved by and conducted in accordance with the regulations of the local Animal Welfare authorities (Landesamt für Gesundheit und Verbraucherschutz, Abteilung Lebensmittel- und Veterinärwesen, Saarbrücken).

The determined plasma concentrations are shown in Figure S6, the pharmacokinetic data are summarized in Table S4.

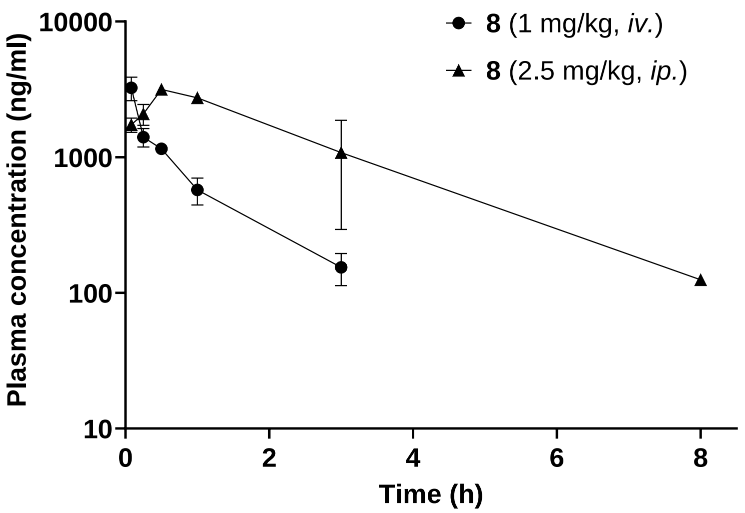

Figure S6. Mean profile of plasma concentrations of the inhibitor **8** after *iv.* (n=3) and *ip.* (n=2) treatment (the lower limit of quantification was set to 120 ng/ml). A plasma level of 1000 ng/ml corresponds to a concentration of approximately 1.3 µM.

Table S4. Pharmacokinetic parameters in male Sprague Dawley rats after treatment with inhibitor **8**. The pharmacokinetic analysis was performed by applying a non-compartment model using the Kinetica 5.0 software (Thermo Scientific, Waltham, USA).

Number of test animals	3	2
Dose route	*iv.*	*ip.*
Dosage (mg/kg)	1	2.5
Volume (mL/kg)	5	5
C_{max} (ng/mL)	-	3156
C_0 (ng/mL)	2076	-
t_{max} (h)	-	0.50
C_z (ng/mL)	154 (after 3h)	126 (after 8h)
t_z (h)	3	8
$t_{1/2z}$ (h)	0.9	1.6
AUC_{0-tz} (ng·h/mL)	2088	9352
$AUC_{0-\infty}$ (ng·h/mL)	2291	9642
V_z (mL/kg)	569	
CL (mL/(h·kg))	442	
$V_{z/f}$ (mL/kg)		639
CL/f (mL/(h·kg))		273

Parameters abbreviations:

C_{max} (ng/mL)	maximal concentration
C_0 (ng/mL)	extrapolated initial concentration
t_{max} (h)	time to reach the maximum concentration
C_z (ng/mL)	last analytically quantifiable concentration
t_z (h)	time of the last sample which has an analytically quantifiable concentration
$t_{1/2z}$ (h)	half life of the terminal slope of a concentration-time curve
AUC $_{(0-tz)}$ (ng·h/mL)	area under the concentration-time curve up to the time t_z of the last sample
AUC $_{(0-\infty)}$ (ng·h/mL)	area under the concentration-time curve extrapolated to infinity
V_z (mL/kg)	volume of distribution, in case of intravascular administration
CL (mL/(h·kg))	total body clearance, in case of intravascular administration
$V_{z/f}$ (mL/kg)	volume of distribution, in case of extravascular administration
CL/f (mL/(h·kg))	total body clearance, in case of extravascular administration

11. Abbreviations used in the supporting information

Amba, 4-amidinobenzylamide; Cav, canavanine; 2-CTC, 2-chlorotrityl chloride; DCM, dichloromethane; DIPEA, N,N-diisopropylethylamine; DMF, N,N-dimethylformamide; HBTU, 3-[Bis(dimethylamino)methyliumyl]-3H-benzotriazol-1-oxide hexafluorophosphate; HOBt, N-hydroxybenzotriazole; *ip.*, intraperitoneal; *iv.*, intravenous; MOI, multiplicity of infection; PyBOP, (Benzotriazol-1-yloxy)tripyrrolidinophosphonium hexafluorophosphate; RMSD, root mean square deviation; RSV, respiratory syncytial virus; SPPS, solid-phase peptide synthesis; TFA, trifluoroacetic acid; TIS, triisopropylsilane, Tle, *tert*-leucine.

12. References

1. Ivanova, T.; Hardes, K.; Kallis, S.; Dahms, S. O.; Than, M. E.; Künzel, S.; Böttcher-Friebertshäuser, E.; Lindberg, I.; Jiao, G. S.; Bartenschlager, R.; Steinmetzer, T., Optimization of Substrate-Analogue Furin Inhibitors. *ChemMedChem* **2017,** *12* (23), 1953-1968.

2. Bernatowicz, M. S.; Wu, Y.; Matsueda, G. R., Urethane protected derivatives of 1-guanylpyrazole for the mild and efficient preparation of guanidines. *Tetrahedron Letters* **1993,** *34* (21), 3389-3392.

3. Becker, G. L.; Sielaff, F.; Than, M. E.; Lindberg, I.; Routhier, S.; Day, R.; Lu, Y.; Garten, W.; Steinmetzer, T., Potent inhibitors of furin and furin-like proprotein convertases containing decarboxylated P1 arginine mimetics. *J Med Chem* **2010,** *53* (3), 1067-75.

4. Gottlieb, H. E.; Kotlyar, V.; Nudelman, A., NMR Chemical Shifts of Common Laboratory Solvents as Trace Impurities. *J Org Chem* **1997,** *62* (21), 7512-7515.

5. Dahms, S. O.; Hardes, K.; Becker, G. L.; Steinmetzer, T.; Brandstetter, H.; Than, M. E., X-ray structures of human furin in complex with competitive inhibitors. *ACS Chem Biol* **2014,** *9* (5), 1113-1118.

6. Dahms, S. O.; Hardes, K.; Steinmetzer, T.; Than, M. E., X-ray Structures of the Proprotein Convertase Furin Bound with Substrate Analogue Inhibitors Reveal Substrate Specificity Determinants beyond the S4 Pocket. *Biochemistry* **2018,** *57* (6), 925-934.

7. Dahms, S. O.; Arciniega, M.; Steinmetzer, T.; Huber, R.; Than, M. E., Structure of the unliganded form of the proprotein convertase furin suggests activation by a substrate-induced mechanism. *Proc Natl Acad Sci U S A* **2016,** *113* (40), 11196-11201.

8. Theveneau, P.; Baker, R.; Barrett, R.; Beteva, A.; Bowler, M. W.; Carpentier, P.; Caserotto, H.; Sanctis, D. d.; Dobias, F.; Flot, D.; Guijarro, M.; Giraud, T.; Lentini, M.; Leonard, G. A.; Mattenet, M.; McCarthy, A. A.; McSweeney, S. M.; Morawe, C.; Nanao, M.; Nurizzo, D.; Ohlsson, S.; Pernot, P.; Popov, A. N.; Round, A.; Royant, A.; Schmid, W.; Snigirev, A.; Surr, J.; Mueller-Dieckmann, C., The Upgrade Programme for the Structural Biology beamlines at the European Synchrotron Radiation Facility – High throughput sample evaluation and automation. *Journal of Physics: Conference Series* **2013,** *425* (1), 012001.

9. Helmholtz-Zentrum Berlin für Materialien und Energie. (2016). The MX beamlines BL14.1-3 at BESSY II. Journal of large-scale research facilities, 2, A47. http://dx.doi.org/10.17815/jlsrf-2-64.

10. Kabsch, W., XDS. *Acta crystallographica. Section D, Biological crystallography* **2010,** *66* (Pt 2), 125-32.

11. Sparta, K. M.; Krug, M.; Heinemann, U.; Mueller, U.; Weiss, M. S., XDSAPP2.0. *Journal of Applied Crystallography* **2016,** *49* (3), 1085-1092.

12. Winn, M. D.; Ballard, C. C.; Cowtan, K. D.; Dodson, E. J.; Emsley, P.; Evans, P. R.; Keegan, R. M.; Krissinel, E. B.; Leslie, A. G.; McCoy, A.; McNicholas, S. J.; Murshudov, G. N.; Pannu, N. S.; Potterton, E. A.; Powell, H. R.; Read, R. J.; Vagin, A.; Wilson, K. S., Overview of the CCP4 suite and current developments. *Acta Crystallogr D Biol Crystallogr* **2011,** *67* (Pt 4), 235-42.

13. Emsley, P.; Lohkamp, B.; Scott, W. G.; Cowtan, K., Features and development of Coot. *Acta crystallographica. Section D, Biological crystallography* **2010,** *66* (Pt 4), 486-501.

14. Adams, P. D.; Afonine, P. V.; Bunkoczi, G.; Chen, V. B.; Davis, I. W.; Echols, N.; Headd, J. J.; Hung, L. W.; Kapral, G. J.; Grosse-Kunstleve, R. W.; McCoy, A. J.; Moriarty, N. W.; Oeffner, R.; Read, R. J.; Richardson, D. C.; Richardson, J. S.; Terwilliger, T. C.; Zwart, P. H., PHENIX: a comprehensive Python-based system for macromolecular structure solution. *Acta crystallographica. Section D, Biological crystallography* **2010,** *66* (Pt 2), 213-21.

15. Schuttelkopf, A. W.; van Aalten, D. M., PRODRG: a tool for high-throughput crystallography of protein-ligand complexes. *Acta Crystallogr D Biol Crystallogr* **2004,** *60* (Pt 8), 1355-63.

16. Hardes, K.; Becker, G. L.; Lu, Y.; Dahms, S. O.; Köhler, S.; Beyer, W.; Sandvig, K.; Yamamoto, H.; Lindberg, I.; Walz, L.; von Messling, V.; Than, M. E.; Garten, W.; Steinmetzer, T., Novel furin inhibitors with potent anti-infectious activity. *ChemMedChem* **2015,** *10* (7), 1218-31.

17. Kacprzak, M. M.; Peinado, J. R.; Than, M. E.; Appel, J.; Henrich, S.; Lipkind, G.; Houghten, R. A.; Bode, W.; Lindberg, I., Inhibition of furin by polyarginine-containing peptides: nanomolar inhibition by nona-D-arginine. *J Biol Chem* **2004,** *279* (35), 36788-94.

18. Williams, J. W.; Morrison, J. F., The kinetics of reversible tight-binding inhibition. *Methods Enzymol* **1979,** *63*, 437-67.

19. Hinkes, S.; Wuttke, A.; Saupe, S. M.; Ivanova, T.; Wagner, S.; Knörlein, A.; Heine, A.; Klebe, G.; Steinmetzer, T., Optimization of cyclic plasmin inhibitors: from benzamidines to benzylamines. *J Med Chem* **2016,** *59* (13), 6370-86.

20. Meyer, D.; Sielaff, F.; Hammami, M.; Böttcher-Friebertshäuser, E.; Garten, W.; Steinmetzer, T., Identification of the first synthetic inhibitors of the type II transmembrane serine protease TMPRSS2 suitable for inhibition of influenza virus activation. *Biochem J* **2013,** *452* (2), 331-343.

21. Walsmann, P., On the purification of thrombin preparations. *Pharmazie* **1968,** *23* (7), 401-402.

22. Van Lam van, T.; Ivanova, T.; Hardes, K.; Heindl, M. R.; Morty, R. E.; Böttcher-Friebertshäuser, E.; Lindberg, I.; Than, M. E.; Dahms, S. O.; Steinmetzer, T., Design, Synthesis, and Characterization of Macrocyclic Inhibitors of the Proprotein Convertase Furin. *ChemMedChem* **2019,** *14* (6), 673-685.

23. Matrosovich, M.; Matrosovich, T.; Garten, W.; Klenk, H. D., New low-viscosity overlay medium for viral plaque assays. *Virol J* **2006,** *3*, 63.

24. Nitsche, C.; Zhang, L.; Weigel, L. F.; Schilz, J.; Graf, D.; Bartenschlager, R.; Hilgenfeld, R.; Klein, C. D., Peptide-Boronic Acid Inhibitors of Flaviviral Proteases: Medicinal Chemistry and Structural Biology. *J Med Chem* **2017,** *60* (1), 511-516.

25. Kouretova, J.; Hammamy, M. Z.; Epp, A.; Hardes, K.; Kallis, S.; Zhang, L.; Hilgenfeld, R.; Bartenschlager, R.; Steinmetzer, T., Effects of NS2B-NS3 protease and furin inhibition on West Nile and Dengue virus replication. *J Enzyme Inhib Med Chem* **2017,** *32* (1), 712-721.

26. Nitsche, C.; Schreier, V. N.; Behnam, M. A.; Kumar, A.; Bartenschlager, R.; Klein, C. D., Thiazolidinone-peptide hybrids as dengue virus protease inhibitors with antiviral activity in cell culture. *J Med Chem* **2013,** *56* (21), 8389-403.

27. Weigel, L. F.; Nitsche, C.; Graf, D.; Bartenschlager, R.; Klein, C. D., Phenylalanine and Phenylglycine Analogues as Arginine Mimetics in Dengue Protease Inhibitors. *J Med Chem* **2015,** *58* (19), 7719-33.
